# NMR-based newborn urine screening for optimized detection of inherited errors of metabolism

**DOI:** 10.1038/s41598-019-49685-x

**Published:** 2019-09-10

**Authors:** Nieves Embade, Claire Cannet, Tammo Diercks, Rubén Gil-Redondo, Chiara Bruzzone, Sara Ansó, Lourdes Román Echevarría, M. Mercedes Martinez Ayucar, Laura Collazos, Blanca Lodoso, Eneritz Guerra, Izaskun Asla Elorriaga, Miguel Ángel Kortajarena, Alberto Pérez Legorburu, Fang Fang, Itziar Astigarraga, Hartmut Schäfer, Manfred Spraul, Oscar Millet

**Affiliations:** 1 Protein Stability and Inherited Disease Laboratory, CIC bioGUNE, Bizkaia Technology Park, Bld. 800, 48160 Derio, Bizkaia Spain; 2 Bruker Biospin GmbH, Silberstreifen, 76287 Rheinstetten, Germany; 3 NMR Platform, CIC bioGUNE, Bizkaia Technology Park, Bld. 800, 48160 Derio, Bizkaia Spain; 4 Department of Pediatrics, Cruces Universitary Hospital, Barakaldo, Spain; 5 Department of Pediatrics, Txagorritxu Hospital, Vitoria-Gasteiz, Spain; 6 Department of Nursing, University of the Basque Country, Donostia, Gipuzkoa Spain; 7 Department of Pediatrics, Basurto Universitary Hospital, Bilbao, Spain

**Keywords:** Predictive markers, Diagnostic markers, Metabolic disorders

## Abstract

Inborn errors of metabolism (IEMs) are rare diseases produced by the accumulation of abnormal amounts of metabolites, toxic to the newborn. When not detected on time, they can lead to irreversible physiological and psychological sequels or even demise. Metabolomics has emerged as an efficient and powerful tool for IEM detection in newborns, children, and adults with late onset. In here, we screened urine samples from a large set of neonates (470 individuals) from a homogeneous population (Basque Country), for the identification of congenital metabolic diseases using NMR spectroscopy. Absolute quantification allowed to derive a probability function for up to 66 metabolites that adequately describes their normal concentration ranges in newborns from the Basque Country. The absence of another 84 metabolites, considered abnormal, was routinely verified in the healthy newborn population and confirmed for all but 2 samples, of which one showed toxic concentrations of metabolites associated to ketosis and the other one a high trimethylamine concentration that strongly suggested an episode of trimethylaminuria. Thus, a non-invasive and readily accessible urine sample contains enough information to assess the potential existence of a substantial number (>70) of IEMs in newborns, using a single, automated and standardized ^1^H- NMR-based analysis.

## Introduction

Inborn errors of metabolism (IEMs) are genetic disorders, usually monogenic disorders, that produce an abnormal accumulation of metabolites due to a malfunctioning protein (i.e. an enzyme or a membrane transporter), involved in the intermediary metabolism. More than 1000 IEMs have been described so far^1^. While each of these disorders may be considered as a rare disease, in total they constitute a significant socio-economic burden with, for instance, an overall incidence of more than 1 in 1.000 affected newborns in Europe. If these diseases remain undetected and untreated, they can lead to irreversible physical and psychological sequels or even death. Thus, IEMs represent a serious public threat that involves intensive, long, and expensive treatments.

While some of these diseases may emerge only in later stages, with chronic and progressive symptoms, most of the neonates with IEMs will develop symptoms typically within hours or days after birth. In this context, an early diagnosis is crucial to adequately prescribe the right therapies to successfully treat these disorders which, in favorable cases, may grant patients a significantly improved quality of life.

Metabolomics is a powerful tool to study IEMs and, for decades, thousands of neonates have been diagnosed through different newborn screening programs. The standard newborn screening card based on blood spots taken from the heel, is routinely analyzed by conventional mass spectrometry^2^. More sophisticated MS-based techniques such as nanospray ionization with high resolution mass spectrometry (nS-HR-MS)^3^ or whole exome sequencing^4,5^ are also available, however they are much more time consuming and/or expensive. NMR spectroscopy is well suited to characterize biofluids as it is a quantifiable, reproducible, non-selective and non-destructive and it is particularly adequate for the characterization of complex solutions (plasma, serum, urine, etc.)^6–8^, although is sensitivity is lower as compared to mass spectrometry. Consequently, NMR has already been applied to neonates with congenital metabolic diseases^9,10^. ^1^H-NMR spectroscopy successfully identified neonates carrying different inborn errors including phenylketonuria^11^, maple syrup urine disease^12^ and errors of purine and pyrimidine metabolism^13^.

In 2014 a group of Turkish hospitals carried out a ^1^H-NMR based clinical study where urine of more than 900 newborns was analyzed to derive the normal concentration ranges for up to 20 normal metabolites and 45 pathological metabolites involved in IEMs^14^. Here, we have implemented and expanded this methodology to conduct a clinical study on urine samples from 470 newborns, obtained from the four public hospitals with neonatology units in the Basque Country. Data analysis allowed to derive the normal metabolic profile of local newborns and to implement an automated screening routine to identify up to 75 congenital metabolic diseases that can be easily extrapolated to other geographical regions.

## Results

### Description of the data collection

Complete demographic data was collected for 470 neonates, except for two samples, with no pertaining metadata. After initial quality control, urine samples from 9 newborns were discarded because they showed centrifugation-resistant turbidity, a feature usually associated with bacterial contamination. The final dataset was composed of 461 samples.

More than 50% of the urine samples were collected at 2–3 days age (Table S1) and all samples were obtained from newborns with a birth weight above 2.5 Kg. The dominant ethnic group is Caucasian (>80%) and males and females were equally represented in the study. Gestational age, head circumference and feeding type varied largely (Table S1).

### ^1^H-NMR spectra acquisition and multivariate analysis

Two different ^1^H-NMR spectra were collected: a high-resolution 1D ^1^H spectrum yielded quantitative metabolite data for statistical analysis and a 2D-*Jres* experiment assisted in peak assignment and metabolite identification. All spectra were collected at 300 K under strict SOPs (see Material and Methods) on either a 600 MHz Bruker AVANCE III (244 samples) or AVANCE IVDr (213 samples) spectrometer, where neither PCA nor PLS analyses revealed any statistical difference between both subsets (Fig. S1).

The 1D ^1^HNMR spectra were then automatically processed and integrated over segments of 0.01 ppm spectral width to obtain bin intensities for statistical analysis. A visual comparison identified six urine samples with poor spectral quality and water suppression that were discarded from further analysis. Using multivariate analysis, 7% of the samples presented multiple regions of the spectra with deviations from normality (Fig. S2) and were no longer considered. This value is lower than previous equivalent studies^11^, and we attribute the discrepancy to the different number of engaged clinical partners and to an improved coordination with the hospitals.

**Figure 1:**
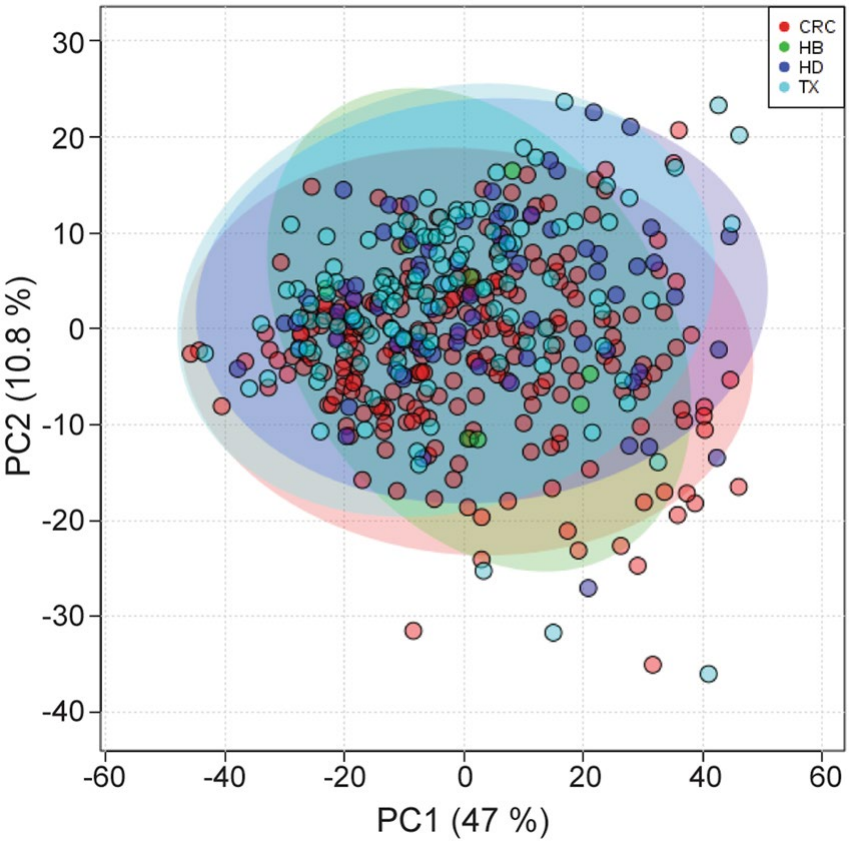
PLS-DA analysis of urine samples. Two-dimensional PLS-DA score plot for different hospitals. HB: Basurto Hospital, CRC: Cruces Hospital, HD: Donosti Hospital, TX: Txagorritxu Hospital.

For the remaining set of 437 urine samples, an initial PCA of bin intensities provided a first survey of the metabolomic data and unbiased rough sample clustering. Neither PCA nor PLS revealed any statistically significant differences between sample subsets from the different hospitals (Fig. 1). Likewise, no correlation with other metadata was observed by PLS-DA except for gender and age at sample collection, for which a metabolic fingerprint is observed (Fig. S3) as reported previously^14^. A slight trend for clustering was observed as a function of head circumference (Fig. S3E), consistent with previous results^15^.

### Targeted analysis: metabolite identification and quantification

Targeted analysis aims at quantifying a given set of metabolites. To that end, the chemical information associated with the bins in the 1D ^1^H-NMR spectrum in combination with the multiplicity information extracted from the 2D J-resolved spectrum was combined to identify up to 150 metabolites in the urine spectrum (Table S2). For each metabolite, the concentration was calculated both, absolute or relative to creatinine. Absolute quantification was referred to the TSP signal in the urine spectra, calculating a signal-intensity per-proton-ratio after the correction for several effects including molecular mass, number of protons, relaxation time, chemical shift, signal multiplicity and coupling constants, line width and Gauss–Lorentz ratio. To that end, signal was fitted using a simplex algorithm also looking for the ranges for signal detection and quantification. Quantification relative to creatinine (in mmol/mol creatinine) used the intensity ratio between a specified metabolite signal and the methyl signal of creatinine.

For a given metabolite, the limit of detection (LOD, Table S2) was determined via spiking experiments from an equivalently measured reference sample database and by simulating spectra after different concentrations of the analyte were added^14^. Averaging over the total cohort allows calculating the probability of obtaining a value above the LOD for the metabolite.

### Probabilistic models for the quantified metabolites

Of 150 metabolites considered, 58 always remained below their LOD (see Table S2) and where, therefore, never detected. Of the remaining 92 metabolites, 66 had a detection incidence of 2.5% or more, allowing to fit a distribution model. As a metabolite’s LOD implies incomplete sampling of the underlying distribution with a natural skew towards higher metabolite concentrations, a generalized extreme value (GEV) distribution model was chosen for its inherent adaptability^16^. GEV is a probability distribution based on extreme value analysis (i. e. extreme deviations from the median of probability distributions) and it seeks to assess the probability of extreme events such as the skewed distributions for the quantified metabolites that considers the LOD values.

Table S2 reports the derived distribution parameters for the 150 metabolites considered and the Q2.5 and Q97.5 quantiles (which delimit the range where 95% of samples are found) obtained from a GEV distribution model (calculated with Eq. 4) or directly from the data histogram. The quantile values obtained by both methods are in excellent agreement except for acetic acid, where the Q97.5 value from the GEV model (332) is larger than that from the data histogram (240) presumably due to the slightly more permissive ranges obtained with the model (Fig. S4).

**Figure 2:**
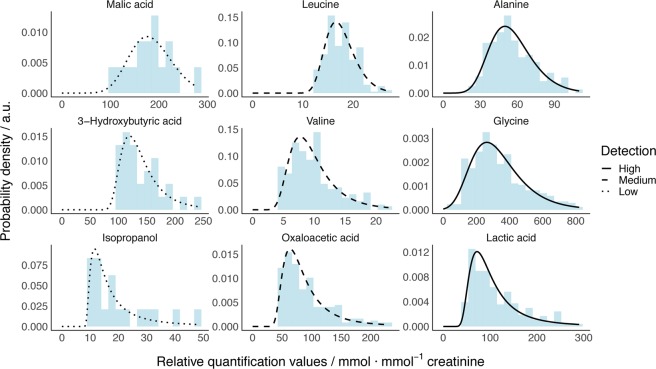
Example of probability densities from 9 representative GEV models. Densities calculated from models are represented as black lines with the following legend: dotted for low, dashed for intermediate and solid for high percentage of detection for the metabolite. Light blue bars plot experimental real data, where values below LOD were discarded.

Figure 2 shows 9 representative examples of metabolite concentration histograms along with modeled probability densities from a GEV distribution that fit well to the experimental data regardless of the detection frequency and the histogram shape (the data for all traceable 66 metabolites with a detection frequency larger than 2.5% is shown in Fig. S4). Thus, the protocol for model building presented here can be applied to derive the normal metabolite concentrations in newborn urine samples for the general population in the Basque Country and to reveal significant deviations as a strong indication for a genetic disorder associated to the pertaining metabolite.

### Diagnostic value of the investigated metabolites

Out of the 150 metabolites determined in the urine samples (Table S2), 7 derive from contaminations from the matrix used (diaper and the plastic consumables), 19 metabolites are associated with bacterial contamination, likely during the sample collection, 14 metabolites derive from drug catabolism (active principles or excipients) and 12 metabolites are associated to the mother’s diet (i. e. caffeine). These metabolites contain little diagnostic nor therapeutic information, but provide metadata information and potential insight on the bacterial gut microbiota of the newborn.

The remaining set of 98 relevant metabolites have been associated with inborn errors of metabolism (IEMs) and, therefore, have potential diagnostic value for the 75 diseases and unhealthy conditions summarized in Table 1. The metabolites are numbered as shown in Table S2. Most commonly, an IEM produces alterations for several metabolites where the “reference metabolites” listed in Table 1 are those required to unambiguously identify an IEM, according to the seminal work of Wevers^17^ and others^18^. Yet, as many metabolites can show abnormal concentrations due to different IEMs, their specific range values (if available, e.g., from HMBD, Metagene) are reported in Table S2. These “support metabolites” are also related to a disorder and can, thus, further confirm an IEM or help to differentiate between related IEMs. Finally, for metabolites that occur in the urine of both healthy and IEM affected newborns, the modeled GEV distributions can be used to determine the probability for our ^1^H NMR based test to indicate a potential IEM case in the Basque Country ($\overline{{F}_{global}}$ listed in Table 1). Such value corresponds to the frequency probability of finding a newborn with a concentration above the pathogenic threshold value. In cases where more than one metabolite showed up with a diagnostic distribution for a given IEM, the larger value of $\overline{{F}_{global}}$ was used for indication.

**Table 1:** IEMs that can be associated to the NMR-based urine analysis.

IEM	Reference metabolites	Support Metabolites	$\overline{{F}_{global}}$
**Organic Acidurias**			
2-ketoadipic acidemia	80, 105, 135		1,82e-6
3-Hydroxy-3-Methylglutaryl-CoA-lyase Deficiency	17, 20, 25	24, 38, 84	1.74e-04
3-Hydroxyisobutiric aciduria	8, 20, 21, 102		n. a.
3-Methyl-crotonyl-glycinuria	20, 24	13	1.61e-3
3-Methyl-glutaconic acidurias	20, 25, 103	54, 132, 135	1.58e-2
Biotinidase deficiency	20, 24, 102	21	2.90e-2
β-Ketothiolase deficiency	25, 143	18, 20, 21, 24, 38	>7.14e-2
Canavan disease	110	135	>7.14e-4
Cobalamin malabsortion	108		4.13e-6
Ethylmalonic encephalopathy	43, 78, 80, 102, 135	12, 114	2.34e-3
Fumaric aciduria	80, 135		7.14e-4
Glutaric aciduria type I	19, 76, 84	18	5.38e-3
Glutaric aciduria type I (low excretor)	19, 76, 84		5.38e-3
Glutaric aciduria type I (non-excretor)	19, 76		n. a.
Glutaric aciduria type II	12, 78, 84, 92	19, 76, 114, 134	2.34e-3
Glutaric aciduria type II (late onset)	20, 78, 84		2.34e-3
Glutaric aciduria type III	84		n.a.
Hyperoxaluria type II	87		>3.09e-1
Isovaleric aciduria	20, 114	12, 18, 86, 108	n. a.
Malonyl-CoA decarboxylase deficiency	78, 108, 135		7.14e-4
Methylmalonic aciduria	16, 75, 91, 129		3.19e-2
Methylmalonate semialdehyde dehydrogenase deficiency	21, 43, 102, 108		2.00e-4
MMA Cbl A deficiency	108	22, 86, 89, 99	4.13e-6
Propionic acidemia	18, 21, 38, 40, 143	3, 22, 86, 89, 99, 128, 129	6.66e-2
Pyroglutamic acidemia	99		n. a.
Transcobalamin II deficiency	108		n. a.
Trimethylaminuria	145		4.25e-3
**Amino Acidurias**			
Argininemia	117, 147	88, 89	4.67e-3
Argininosuccinic aciduria	47, 117, 147	86, 89	4.37e-3
Cystinuria	46, 58		4.01e-5
Dicarboxylic aminoaciduria	82		n. a.
Dimethylglycine dehydrogenase deficiency	49, 115		5.91e-12
Hartnup disease	43, 83, 98, 103, 149	75, 101	4.39e-4
Hawkinsinuria	32, 99		8.54e-2
Holocarboxylase syntetase deficiency	20, 21, 22		2.07e-1
Homocystinuria	97	49, 107	n.a.
Kyneureninase deficiency	74, 150		n. a.
Maple Sirup Urine disease	9, 15, 23	3, 8, 14, 18, 98, 103, 149	8.46e-8
Methionine malabsortion	11		n. a.
Mild Phenylketonuria	116, 124		n. a.
Phenylketonuria	10, 26, 32, 112, 123, 124, 125	37, 65	n. a.
Saccharopinuria	55		n. a.
Sarcosinemia	134		4.56e-1
Tyrosinemia type I	31, 32, 33, 75, 136	35, 113, 147	5.83e-3
Tyrosinemia type II	31, 33	113	3.51e-2
Tyrosinemia type III	31, 32, 33		>7.62e-2
Transient newborn tyrosinemia	31, 33		3.51e-2
Valinemia	149		4.39e-4
**Fatty Acid Oxidation Disorders**			
3-Oxoacid CoA transferase deficiency	18, 38		5.38e-3
Long Chain 3-hydroxyacyl-CoA dehydrogenase deficiency	18		6.66e-2
Short Chain Acyl-CoA dehydrogenase deficiency	12, 78		4.92e-5
Short Chain 3-hydroxyacyl-CoA dehydrogenase deficiency	18		6.66e-2
**Urea Cycle Disorders**			
Carbamoyl Phosphate synthetase I deficiency	86, 99, 147		4.37e-3
Citrullinemia	55, 117, 147, 148	47, 61, 89	3.47e-1
Neonatal intrahepatic cholestasis	32		n. a.
Ornitine carbamoyltransferase deficiency	117, 147, 148	86, 89, 99	4.67e-3
Creatine Deficiencies			
Creatine transport deficiency	56, 57		n. a.
Guanidinoacetate Methyltransferase deficiency	88		n. a.
**Purine/Pyrimidine Disorders**			
Dihydropyrimidine Dehydrogenase deficiency	141, 147		4.37e-3
Dihydropyriminidase deficiency	70, 71	141, 146, 147	4.37e-3
Orotic aciduria	117		n. a.
**Carbohydrate Disorders**			
Fructose-1,6-bisphosphatase Deficiency	18, 38, 40	85	>7.14e-2
Fanconi-Bickel syndrome	63	61, 81	>3.47e-1
Galactosemia	61, 81	60	3.47e-1
**Lysosomal Disorders**			
Tay-Sachs disease	99		n. a.
**Mitochondrial Disorders**			
Dihydrolipoyl dehydrogenase E3	14, 23		2.29e-4
Lactic acidemia	43, 102	21	2.9e-2
Pyruvate carboxylase deficiency	18, 40, 102	43, 135	5.38e-3
**Porphyrias**			
Acute Intermittent Porphyria	35		1.49e-5
Delta-aminolevulinic acid dehydratese deficiency	35		1.49e-5
**Other Disorders**			
Asphyxia	23, 102		4.03e-2
Aminoacylase I deficiency	111		n. a.
GABA transaminase deficiency	27, 55		>1.24e-1
Molybdenum cofactor deficiency	139		1.42e-2
Ketosis	18, 38		6.66e-2

List of different inborn errors of metabolism that can be identified by NMR. Reference metabolites are required to unambiguously identify a given IEM. Support Metabolites add value to confirm a specific IEM and/or help to discriminate between related IEMs. All numbers are related to the metabolites listed in Table S2. $\overline{{F}_{global}}$ describes the probability for the test to identify a potential IEM case in the Basque Country.

### Metabolic disorders detected within the cohort

In at least two newborn urine samples, we found abnormal concentrations of metabolites related to an alteration in the ketonic bodies. The first sample showed concentrations of 3-hydroxybutyric acid (240 mmol/mol creat), acetone, (210 mmol/mol creat), and acetoacetic acid (62 mmol/mol creat) far above their upper limits derived from the modeled distributions, with probabilities of 0.002, 0.0012, and <0.0001, respectively. Moreover, these metabolite concentrations were also higher than states as normal in metabolomics databases (HMDB, Metagene). The second sample likewise showed abnormally high concentrations for 3-hydroxybutyric acid (170 mmol/mol creat, probability 0.0124) and acetoacetic acid (49 mmol/mol creat, probability 0.0004), which were accompanied by an elevated glucose concentration (560 mmol/mol creat, probability 0.0083). Taken together, these experimental results strongly suggest two cases of ketosis that might be related to a transition state of the newborn.

Finally, a third newborn presented high levels of trimethylamine (24 mmol/mol creatinine; probability 0.0014) suggesting the IEM Trimethylaminuria (FMO3) since the normal concentrations in newborns, as determined in our ^1^H NMR study (Fig. 2), are not higher than 6 mmol/mol creat. Yet, this finding should be accompanied by elevated concentrations TMAO, currently unavailable within our method.

## Discussion

In this contribution we have investigated novel strategies for an early identification of congenital metabolopathies from ^1^H NMR data from newborn urine samples. Even though IEMs are individually considered as rare disorders, their collective incidence is considerable and represents a serious public health problem. An early diagnosis of these pathologies is crucial to avoid serious acute symptoms and/or to avoid a lifelong treatment of patients. In the context of personalized medicine, even a negative (unsuspicious) result of such IEM tests still produces relevant data that can be integrated in a personal database together with molecular data which is expected to gain diagnostic value in the future.

In the last years had been increasing the number of IEM (around 50) that can be detected using mass spectrometry^19^, even though that the high sensitivity of the technique suggests that this number will increase. Moreover, MS requires the collection of dried blood spots (DBS), which is done by pricking the heel of newborns. It is easy to perform but the process is in a certain way invasive. In NMR spectroscopy, currently 75 IEMs may be detected and this number is also increasing over time. In here, up to 1000 urine metabolites are analyzed simultaneously in a measurement that avoids pricking and takes only minutes. In summary, compared to other techniques such as MS, NMR spectroscopy has great potential for newborn screening, as it is able to sample the metabolic profile and detect both known and unknown metabolites in a quantitative and non-targeted way. Moreover, it is reproducible, and the quantification of urine metabolites is independent on the instrument configuration as long as the SOPs are followed. Finally, the measurement is economically advantageous since the comprehensive metabolite set is already obtained. All these aspects position the technique in a leading place for a potential implementation of the methodology in hospitals and health centers for the screening of large population segments.

Using the metabolomics data provided from the whole cohort of newborns along with statistical analysis, we were able to create a healthy metabolome profile at a high degree of precision. A subset of 66 of the analyzed metabolites were found present in at least 2.5% of all the newborns. Normal ranges for these common metabolites have been established using automatic quantification, as shown in Figs S4–S6, and Table S2, also compared to the normal and pathogenic levels reported in the literature. Some metabolites showing close proximity (or partial overlap) between normal and pathogenic concentrations reflect a heterogenic origin for the metabolite or an incomplete understanding of the relationship between the metabolite and the disease.

IEM incidence strongly depends on geographical and ethnic background of a population^20^ and it is instructive to compare our model with the Turkish model previously reported^14^. The metabolites found in our study enclose 18 out of the 20 found in the Turkish work, while myo-Inositol and trimethylamine are found very often only in the Turkey model. We attribute such discrepancy to an improved identification of the two metabolites in our deconvolution algorithm.

Another 84 metabolites that are typically not found at NMR detectable level in healthy newborns were validated for their absence. Finally, we were also able to observe metabolites from different sources of contamination. Propylene glycol was present in about half of the urine samples likely from external manipulation. This aliphatic alcohol is a chemical included in cosmetics, skin conditioning also found in medication^21^. Moreover, the metabolite acetoin (present in perfumes) was observed in a few spectra from the same hospital (Fig. S2), while unknown metabolites could be detected in a subset of the spectra as well. A future goal is to proceed with the identification of unknown peaks, to determine whether they contain potentially diagnostic information.

**Figure 3:**
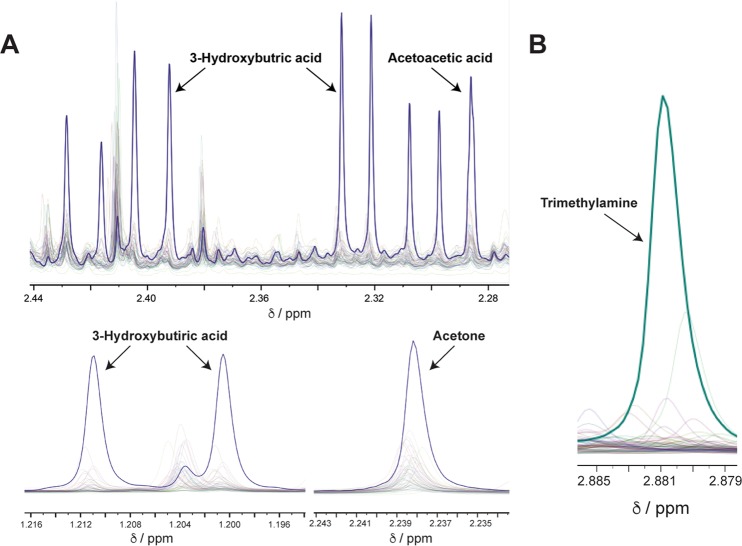
Newborn diseases identify by NMR metabolomics. (**A**) Three different markers (3OH-butyric acid, acetoacetic acid and acetone) in an NMR spectrum of a urine sample from a newborn, show high concentrations as compared to the normal ranges of intensity for other urine samples, suggesting ketosis. (**B**) The metabolite trimethylamine shows also an extremely high concentration in one sample, strongly indicating the presence of trimethylaminuria for that neonate.

Deviations from normality in some of those common metabolites provide very useful information about rare and prevalent metabolic disorders. Up to 75 IEMs may potentially be detected based on the quantification of a single metabolite or the combination of several of them (Table 1). Such disorders can be divided into urea cycle disorders, organic acidurias, purine/pyrimidine disorders, amino acidurias, carbohydrate disorders, creatine deficiencies, fatty acid oxidation disorders and other diseases and syndromes. For example, elevated concentrations of 3-hydroxybutyric acid, acetone and acetoacetic acid revealed a couple of ketosis cases (Fig. 3). Ketosis is a metabolic state where the newborns use fat as fuel in preference to carbohydrates. The body makes ketones from fat, when dietary glucose (from carbohydrates and sugar) is low. Acetoacetate is one of the products of fatty acid oxidation and acetone and hydroxybutyrate are formed from acetoacetate^22^. In one case, the metabolic alteration was accompanied by high levels of glucose, an indicator of Insuline-dependant diabetes Mellitus. In another unrelated sample, a case of trimethylaminuria was also potentially identified. Hence, this approach has proven very useful for the discrimination of pathological samples by identifying abnormal metabolite concentrations or patterns after comparison with the model. Unfortunately, the samples were codified and anonymized so clinicians did not have the opportunity to follow the babies that showed these abnormal metabolites.

The number of samples analyzed constitute a limitation in our study: approximately 1:1000 neonates may be affected by a congenital metabolic disorder worldwide. In this context, analyzing less than 500 samples, we were able to detect up to three different metabolopathies, showing the method’s potential. Yet, a higher number of samples will be needed to further validate the analytical technique.

In summary, the characterization of a large set of samples prompted us to develop a statistical model for several metabolites applicable to the healthy Basque Country newborn population. The model allows the identification of 75 different IEMs based on the comparative analysis of the absolute concentration of metabolites from a test sample in the context of the normal values obtained by the model which, in correlation with clinical parameters, allows the diagnosis and eventually the monitoring of patients throughout their illness^23^. The reasonable agreement with the number of metabolites and the concentration ranges found in other regions (i. e. Turkey) underline the idea that the model can be easily extrapolated to multiple regions, ultimately constituting a reliable alternative to the heel test for IEMs premature detection.

## Methods

### Study design

Urine samples were collected from 470 individuals that were born in one of the four public hospitals of the Basque Country with neonatology units: Cruces (Barakaldo, Bizkaia), Txagorritxu (Vitoria, Araba), Basurto (Bilbao, Bizkaia) & Donostia (Donostia, Guipuzkoa). All samples were codified and anonymized to protect the confidentiality of individual participants.

### NMR measurements

For each sample, a one-dimensional (1D) ^1^H-NMR spectrum with water peak suppression^24^, and a 2D *J*res experiment were collected at 300 K, using the Standard Operating Procedures described in the Supplementary Materials and Methods.

### Statistical analysis

Outliers were properly identified and eliminated from the dataset. Multivariate and univariate statistics was applied to the dataset as explained in the Supplementary Materials and Methods.

### Generalized extreme value distributions

The GEV distributions can be described by three parameters (location μ, scale σ, and shape ξ) and provide different levels of information. As usual in probability distributions, the cumulative distribution function (CDF) gives the probability of obtaining a value (concentration in this case) that is lower or equal than a specific threshold x. The CDF (F_GEV_) for a GEV distribution can be calculated according to the following expression

1 $${F}_{GEV}(s;\xi )=\{\begin{array}{ll}\exp (\,-\,{(1+\xi s)}^{-1/\xi }) & \xi \ne 0\\ \exp (\,-\,\exp (-\,s)) & \xi =0\end{array}$$

where s is the scaled value of x (s = (x − μ)/σ). Similarly, it is possible to calculate the probability of obtaining a concentration higher than x using the complementary CDF ($\overline{{F}_{GEV}}=1-{F}_{GEV}$). Due to LOD, F_GEV_ was in fact incomplete because the fraction of samples below LOD had to be considered and the global CDF (F_global_) was calculated with the following expression

2 $${F}_{global}=(1-\lambda )+\lambda {F}_{GEV}(s;\xi )$$

where λ is the fraction of samples above LOD. From this expression is straightforward to deduce that

3 $$\overline{{F}_{global}}=\lambda \overline{{F}_{GEV}}(s;\xi )$$

Moreover, since the CDF is invertible it is also possible to calculate specific concentrations associated to quantiles using function Q_GEV_, as shown in Eq. 4:

4 $$Q(p;\mu ,\sigma ,\xi )=\{\begin{array}{ll}\mu +\sigma ({(-\log (p))}^{-\xi }-\,1)/\xi  & \xi  > 0\,{\rm{and}}\,p\in [0,1);\,\xi  < 0\,{\rm{and}}\,p\in (0,1]\\ \mu -\sigma \,\log (\,-\,\log (p)) & \xi =0,\,p\in (0,1),\end{array}$$

where p is the quantile for which is wanted to obtain its associated concentration. It is also affected by incompleteness of CDF due to LOD. For this reason, quantile q must be adjusted as follows:

5 $$p^{\prime} =\frac{p-(1-\lambda )}{\lambda }$$

where the numerator calculates the amount of quantile p that is not covered by the fraction below LOD (1 − λ) and the numerator scales this result in the context of the modeled GEV.

### Ethical approval

All procedures followed were in accordance with the Helsinki Declaration of 1975 and under the guidance of the Good Clinical Practice (GCP). The study was approved by the Basque Country’s Clinical Research Ethics Committee (CEIC) with the title “Metabolomic analysis of newborn urine for the improvement of the diagnosis of congenital pathologies”. Informed consent was obtained from parents of all the newborns included in the study. All samples were codified and anonymized to protect the confidentiality of individual participants. The sample collection and the measurement of the urines by NMR was carried out from 2015 to 2017.

## Supplementary information

Supplementary Material
