# Supplementary material for: NMR-based newborn urine screening for optimized detection of inherited errors of metabolism
Source: Sci Rep. 2019 Sep 10;9:13067. doi: 10.1038/s41598-019-49685-x (PMC6736868; doi:10.1038/s41598-019-49685-x)
Supplement: Supplementary file 1 — Supplementary Material [file 41598_2019_49685_MOESM1_ESM.pdf]

Supplementary material for:

NMR-based newborn urine screening for an optimized premature detection of inherited errors of metabolism

Nieves Embade[1], Claire Cannet[2], Tammo Diercks[3], Rubén Gil-Redondo[1], Chiara Bruzzone[1], Sara Ansó[4], Lourdes Román Echevarría[4], M. Mercedes Martinez Ayucar[5], Laura Collazos[5], Blanca Lodoso[5], Eneritz Guerra[4], Izaskun Asla Elorriaga[4], Miguel Ángel Kortajarena[6], Alberto Pérez Legorburu[7], Fang Fang[2], Itziar Astigarraga[4], Hartmut Schäfer[2], Manfred Spraul[2], and Oscar Millet[1,*]

[1]Protein Stability and Inherited Disease Laboratory, CIC bioGUNE, Bizkaia Technology Park, Bld. 800, 48160 Derio, Bizkaia, Spain.

[2]Bruker Biospin GmbH, Silberstreifen, 76287 Rheinstetten , Germany.

[3]NMR Platform, CIC bioGUNE, Bizkaia Technology Park, Bld. 800, 48160 Derio, Bizkaia, Spain.

[4]Department of Pediatrics, Cruces Universitary Hospital, Barakaldo, Spain.

[5]Department of Pediatrics, Txagorritxu Hospital, Vitoria-Gasteiz, Spain.

[7]Department of Nursing, University of the Basque Country, Donostia, Gipuzkoa, Spain.

[8]Department of Pediatrics, Basurto Universitary Hospital, Bilbao, Spain.

Contents:

Supplementary Materials and Methods.

Table S1. Clinical metadata of the Studied Newborn Population.

Figure S1. PCA analysis for AVANCE 600 MHz spectrometer versus IVDr 600 spectrometer in urine samples.

Figure S2. Different types of metabolites found out in the spectra.

Figure S3. Two-dimensional PLS-DA score plot urine samples.

Table S2. LOD values, GEV distribution values, quartiles, normal and pathogenic values for the 150 metabolites under consideration.

Figure S4. Distribution of probability densities for 22 out of the 66 metabolites that had a percentage of detection greater or equal 2.5%.

Figure S5. Distribution of probability densities for 22 out of the 66 metabolites that had a percentage of detection greater or equal 2.5%.

Figure S6. Distribution of probability densities for 22 out of the 66 metabolites that had a percentage of detection greater or equal 2.5%.

Supplementary Materials and Methods

Study design. Urine samples were collected from 470 individuals that were born in one of the four public hospitals of the Basque Country with neonatology units: Cruces (Barakaldo, Bizkaia), Txagorritxu (Vitoria, Araba), Basurto (Bilbao, Bizkaia) and Donostia (Donostia, Guipuzkoa). Samples were codified and anonymized to protect the confidentiality of individual participants. Metadata such as date of birth, gestational age, gender, weight, length, head circumference, newborn lifetime at sampling and feeding type was included for the majority of the donors. Neonates, with clinical symptoms of any disease were not considered in the study.

To minimize variation and to achieve maximal reproducibility, we followed strict standard operating protocols (SOPs) for the whole duration of the study. All materials used in the hospitals for urine collection and handling belonged to the same brand and lot number. Urine was collected from cotton balls inserted into the diapers which were subsequently extracted from the cotton balls by placing them into the syringe barrel, inserting the plunger and squeezing out the absorbed urine into a 15 mL Falcon tube (Roberts and Lucas 1985). The obtained urine was aliquoted into 1.5 mL Eppendorf tubes and stored at −80 °C until NMR analysis. To avoid contamination of material from diapers or cotton ball (Goodpaster et al 2011) we also collected samples from diapers and cotton balls, soaked with water for different times (1h to 6h) and temperatures (RT versus 37ºC). The absorbed liquid samples were measured following the same procedure as for the newborn urine.

NMR sample Preparation. On the day of analysis, frozen urine samples were left to thaw to room temperature for 30 min and then centrifuged at 6000 rpm for 5 min at 4°C. An aliquot of 630 µL was transferred into a 1.5 mL microcentrifuge tube without swirling up any sediment from the bottom of the original tube to avoid contamination of the NMR sample by sedimentation, which might occur during centrifugation. Human urine covers a broad pH range and contains a variable amount of ionic species that can affect the NMR chemical shifts of metabolites. To minimize the pH variation, we added 70 µL of a phosphate buffer (1.5 M KH_2PO_4/K_2HPO_4, 2 mM NaN_3, 1% TSP in 70% D_2O, pH 7.4) to thes amples that were then briefly vortexed. Finally, 600 µL of each urine/buffer mixture was pipetted into a 5 mm Wilmad 507-PP-7 NMR tube with specified camber and excentricity (for high-resolution shimming) as well as sampled volume (for quantification).

NMR measurements. Samples were measured on two 600 MHz BRUKER spectrometers, both equipped with ^1H (inverse) detecting room temperature probeheads with automatic tuning and matching unit: i) an AVANCE III with PA-TXI probehead with triple axis gradient coils and an automatic *SampleXpress* sample changer operating at room temperature (296 K), or ii) an AVANCE IIIHD (IVDr) with PA-BBI probehead with z-gradient coil and an automatic *SampleJet* sample changer operating at 278 K. All urine samples were prepared manually at 277 K in batches of 45 at a time to minimise formation of a concentration gradient inside the NMR tubes stored in the automatic sample changer (with a sampling rate of 3 – 4 per hour, this corresponded to maximal 12 hours waiting time for the last NMR sample loaded). These were then prepared for NMR measurements by the following automated steps: 1) loading the NMR tube into the magnet (with prior warming to 300 K in the SampleJet), 2) temperature (300 K) stabilisation during 5 minutes, 3) locking on the D_2O signal, 4) LOCK phase optimisation (only on the IVDr spectrometer), 5) ^1H tuning and matching, 6) gradient shimming (z to z5), 7) ^1H 90° pulse length and 50 Hz presaturation power calibration (using a fast nutation experiment). As described below, two ^1H NMR experiments were then measured per urine sample, each with selective water presaturation (50 Hz saturation power) during the interscan delay.

A high-resolution 1D ^1H spectrum (BRUKER pulse sequence '*noesyphpr1d*', (Mackay 2011)) was acquired with 4 dummy (DS) and 32 accumulated (NS) scans, 4 s interscan delay (D1), 20 ppm spectral width (SW), 64K complex data points (TD) resulting in 2.7 s FID acquisition time (AQ) and 0.367 Hz FID resolution (1/AQ). The FID was then apodised with an exponential function (0.3 Hz linebroadening factor) and zero filled to 128K data points (SI) prior to Fourier transform. The obtained spectrum was automatically phase and baseline corrected, and referenced to the internal TSP signal (at 0.00 ppm). Finally, an artificial PULCON reference signal with an amplitude corresponding to 1 mM ^1H concentration was added at 12 ppm (Wider and Dreier 2006) to enable a quantitative spectrum analysis.

A 2D J_{HH} resolved spectrum (BRUKER pulse sequence '*jresgpprqf*') was acquired with DS = 16, NS = 2, D1 = 2 s, SW = 16.7 ppm, TD = 8K resulting in AQ = 0.41 s and 2.45 Hz FID resolution; the indirect J_{HH} dimension was sampled with a spectral width of 78 Hz and 40 data points (serial FIDs) resulting in 1.85 Hz resolution. The serial FIDs were then multiplied with a sine function for resolution enhancement and zero filled to SI = 16K in the direct and 256 in the indirect dimension prior to Fourier transform (in both dimensions). The obtained spectrum was processed in magnitude mode and tilted (45°) to construct a homodecoupled ^1H spectrum by projection in the direct dimension.

Critical experimental parameters were verified and calibrated daily, prior to any screening experiment, using commercially available NMR standards. Thus, the NMR sampling temperature was calibrated to 300 ± 0.1 K using a 99.8% deuterated pure methanol sample. The magnetic field homogeneity ('shim quality') was verified using a standard aqueous sucrose sample (2 mM, 4 cm filling height), and readjusted if the ^1H signal linewidth of the included TMS was larger than 0.6 Hz (processed without FID apodisation). Using the same standard sample, the H_2O signal offset was calibrated by minimising the residual H_2O signal after 10 s presaturation at 50 Hz power, and the water suppression quality was verified using the SUPPCAL routine (BRUKER Biospin). Finally, an external quantification reference sample (QuantRef, BRUKER Biospin) was used to verify the stability of signal intensities (within ±2%

tolerance) and, if required, perform a recalibration for absolute spectra quantification by the PULCON principle (Wider and Dreier 2006).

Metabolite quantification and determination of LODs. Metabolite quantification was performed after spectra deconvolution and signal fitting using an in-house built simplex algorithm. The method is similar to the commercially available solutions and considers all the chemical and topological parameters that are constitutive of the metabolite: chemical shift, signal multiplicity due to homonuclear J-coupling, relative intensity due to the number of contributing protons and linebroadening due to relaxation. In addition, it also considers the Gauss/Lorentz ratio to adjust to the processing parameters. Gausian/lorentzian synthetic shapes were tested on the complex envelope function to fit the concentration of the metabolite. Fit examples for illustration of automated quantification approach in urine spectra can be found in Aygen *et al*. (Aygen et al 2014).

The limits-of-detection (LOD) for the different metabolites were determined as previously reported (REF) by using spiking experiments at growing concentrations of the reference metabolite. This procedure was repeated for all the samples in a representative set of at least 10 urines that captures the intrinsic variability of the urine sample.

Untargeted analysis. The ^{1}H-NMR spectra dataset, processed using Topspin (Bruker Biospin), was further analyzed using MestreNova software (v.7.0, Mestrelab Research S.L) and R (v.2.3-3, the R project for Statistical Computing). Each spectrum was segmented into consecutive buckets (bins) of fixed 0.01 ppm width in the region between 9.5 and 0.5 ppm and pertaining bin intensities were computed by dividing the bin integral by the bin width. Bins in the regions 4.5 to 5.0 ppm (residual water signal) and 5.4 to 6.0 (urea signal) were discarded, leaving a total of 790 bins ([9.5 – 0.5 – (5.0 – 4.5) – (6.0 – 5.4)] ppm / 0.01 ppm) for analysis. Bin intensities were then normalized to pertaining creatinine integral in order to minimize the effect of different urine concentrations, and subsequently *Pareto* scaled using MetaboAnalyst 4.0 (http://www.metaboanalyst.ca) (Chong et al 2018) to reduce bias from sample variability and preparation. No bin alignment was performed in any case.

Targeted analysis and metabolic model build-up. Concentrations for each metabolite were fitted to a generalized extreme value (GEV) distribution using a maximum-likelihood estimation (MLE) algorithm with Broyden–Fletcher–Goldfarb–Shanno (BFGS) optimization, as implemented in the "evd" R package (v.2.3-3). The number of available quantified values was small for some metabolites, while for others the modes were concentrated in very few discrete values, both of which reduces the MLE accuracy. Therefore, a bootstrapping strategy was implemented in combination with small random variation of the data: For each metabolite, 100 MLE calculations were performed, each with a different subset of 1000 values obtained from random sampling with replacement. A small random variation was applied to each value, between 0 and 0.2 times the minimum difference between 2 consecutive values. Finally, scale, location, and shape parameters were averaged over the 100 MLE casts.

Sample outlier identification. A combination of *multivariate* and *univariate* statistics was applied to classify samples as outliers. The multivariate analysis, based on DBSCAN (Ester et al 1996), used multivariate space (bins, corresponding to spectral sections of 0.01 ppm width) to identify sample groups with low density or isolated samples, marked then as extremes. The univariate analysis independently identified outlier bins and samples with at least 20% outlier bins were then marked as extremes. Input parameters for the DBSCAN clustering algorithm were set to 35 for the size of the epsilon neighborhood (eps) and to 50 for the number of minimum points in the eps region (minPts). In the univariate bin analysis, outlier values were assigned by the 1.5-times-interquartile-range (1.5IQR) rule, i.e. values greater than Q3 + 1.5IQR or lower than Q1-1.5IQR were marked as outlier.

References

Aygen S, Durr U, Hegele P, et al (2014) NMR-Based Screening for Inborn Errors of Metabolism: Initial Results from a Study on Turkish Neonates. *JIMD Rep* 16: 101-111.

Chong J, Soufan O, Li C, et al (2018) MetaboAnalyst 4.0: towards more transparent and integrative metabolomics analysis. *Nucleic Acids Res* 46: W486-W494.

Ester M, Kriegel HP, Sander J, Xu X (1996) A density-based algorithm for discovering clusters in large spatial databases with noise. *Proc Discov Data Mining*: 226-231.

Goodpaster AM, Ramadas EH, Kennedy MA (2011) Potential effect of diaper and cotton ball contamination on NMR- and LC/MS-based metabonomics studies of urine from newborn babies. *Analytical chemistry* 83: 896-902.

Mackay RT (2011) How the 1D-NOESY suppresses solvent signal in metabolomics NMR spectroscopy: an examination of the pulse sequence components and evolution. *Concepts in Magnetic Resonance Part A* 38A: 197-200.

Roberts SB, Lucas A (1985) Measurement of urinary constituents and output using disposable napkins. *Arch Dis Child* 60: 1021-1024.

Wider G, Dreier L (2006) Measuring protein concentrations by NMR spectroscopy. *Journal of the American Chemical Society* 128: 2571-2576.

Table S1. Clinical metadata of the Studied Newborn Population.

Gender		Male	Female
**Number of samples**		242	213
**Hospital**	CRC	134 (55.37%)	114 (53.52%)
	HB	3 (1.24%)	5 (2.35%)
	HD	33 (13.64%)	40 (18.78%)
	TX	72 (29.75%)	54 (25.35%)
**Ethnic group**	Asian	2 (0.83%)	2 (0.94%)
	Caucasian	202 (83.47%)	176 (82.63%)
	Hispanic	26 (10.74%)	24 (11.27%)
	Maghreb	10 (4.13%)	8 (3.76%)
	Black	1 (0.41%)	3 (1.41%)
	'Missing'	1 (0.41%)	0 (0.00%)
**Gestation term**	preterm	4 (1.65%)	1 (0.47%)
	early term	47 (19.42%)	46 (21.60%)
	full term	141 (58.26%)	123 (57.75%)
	late term	49 (20.25%)	42 (19.72%)
	'Missing'	1 (0.41%)	1 (0.47%)
**Weight at birth / g**	low (<3000g)	42 (17.36%)	47 (22.07%)
	normal	200 (82.64%)	166 (77.93%)
**Weight at collection / g**	2000-2500	5 (2.07%)	7 (3.29%)
	2500-3000	61 (25.21%)	63 (29.58%)
	3000-3500	120 (49.59%)	105 (49.30%)
	3500-4000	49 (20.25%)	34 (15.96%)
	4000-4500	6 (2.48%)	1 (0.47%)
	'Missing'	1 (0.41%)	3 (1.41%)
**Food type at collection day**	artificial	25 (10.33%)	23 (10.80%)
	maternal	173 (71.49%)	147 (69.01%)
	mixed	43 (17.77%)	42 (19.72%)
	'Missing'	1 (0.41%)	1 (0.47%)
**Age at collection / days**	1	91 (37.60%)	88 (41.31%)
	2	127 (52.48%)	98 (46.01%)
	3	11 (4.55%)	10 (4.69%)
	4	4 (1.65%)	4 (1.88%)
	'Missing'	9 (3.72%)	13 (6.10%)
**Head Circumference at collection / cm**	30-32	3 (1.24%)	11 (5.16%)
	32-34	71 (29.34%)	93 (43.66%)
	34-36	146 (60.33%)	95 (44.60%)
	36-38	21 (8.68%)	10 (4.69%)
	>38	1 (0.41%)	4 (1.88%)

Legend: Cases with missing value were represented with 'Missing' category. CRC: Cruces Hospital; HB: Basurto Hospital; HD: Donosti Hospital; TX: Txagorritxu Hospital. Preterm: 35-37 weeks; early term: 38-39 weeks; full term: 40 weeks; late term, 41-42 weeks. Weight at born normal >3 Kg. Mixed food type: maternal and artificial milk.

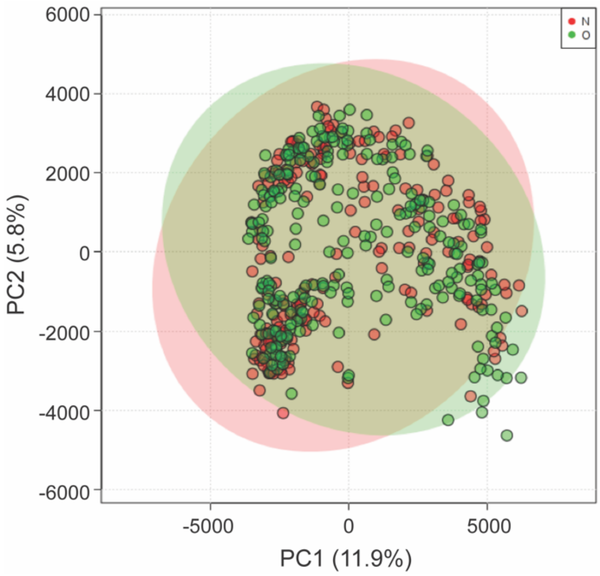

Figure S1. PCA analysis for AVANCE 600 MHz spectrometer versus IVDr 600 spectrometer in urine samples. N: Samples measured in the IVDr 600Mz spectrometer, O: Samples measured in the AVANCE 600 MHz spectrometer.

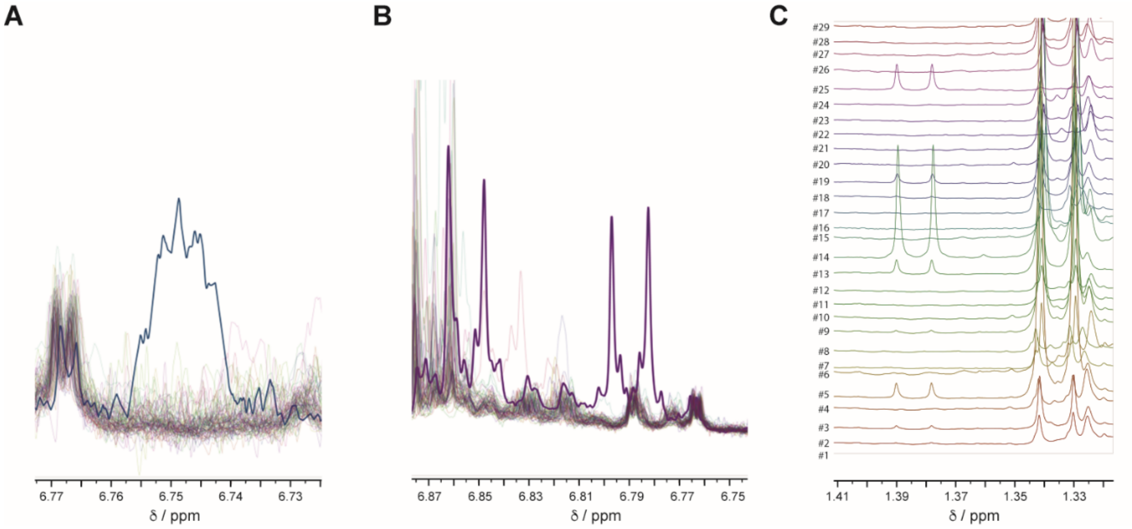

Figure S2. Different types of metabolites found out in the spectra. A) Unknown metabolite; B) 4-Phenylacetic acid at abnormal concentration. Phenylketonuria would also require altered levels of 4-hydroxyphenyllactic acid and 4-hydroxyphenylpyruvic acid, but they were found normal. C) Acetoin presence in several urines (#5, #13, #14, #19 and #25) at disparate concentrations is an example of external contamination.

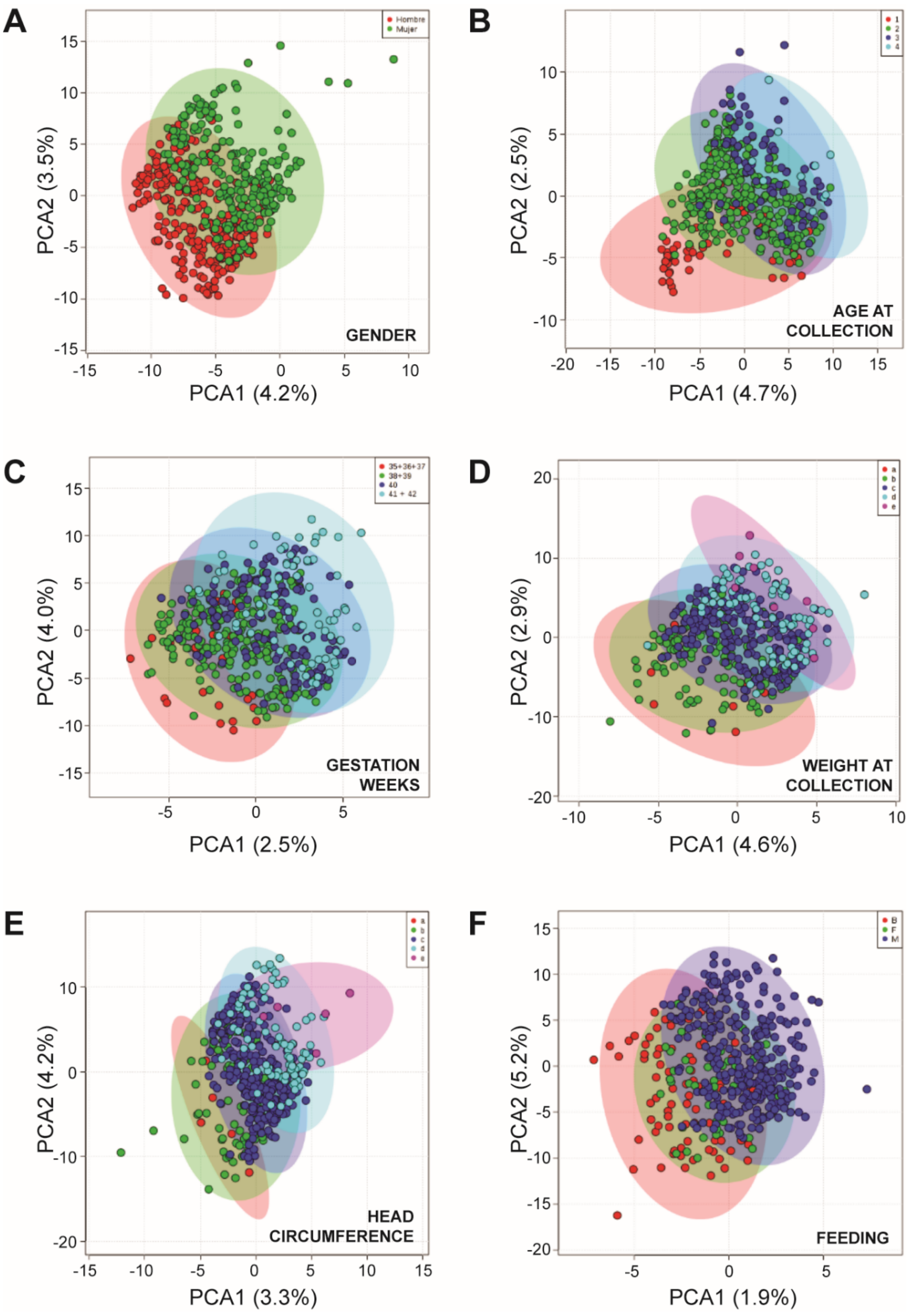

Figure S3. Two-dimensional PLS-DA score plot urine samples. A) Gender, R2= 0.405, Q2= -0.211. B) Newborn age when the urine sample was taken. 1: 1 day; 2:2 days; 3: 3 days; 4: 4 days. R2= 0.465, Q2= - 0.095 C) Gestation weeks: a: preterm, 35-37 weeks; b: early term, 38-39 weeks; c: full term, 40 weeks; d; late term, 41-42 weeks. R2= 0.404, Q2= - 0.097. D) Weight of the newborn in Kg when the sample was taken. a: 2-2.5, b: 2.5-3, c: 3-3.5, d: 3.5-4, e:>4. R2= 0.474, Q2= - 0.213. E) Head circumference in cm, a: 30-32, b: 32-34; c: 34-36; d: 36-38; e: < 38 cm. R2= 0.483, Q2= - 0.235 F: Feeding type, B: Breastfeeding, F: artificial, M: mixed. R2= 0.433, Q2= - 0.183.

Table S2. LOD values, GEV distribution values, quartiles, normal and pathogenic values for the 150 metabolites under consideration.

ReMetabolite		LOD mmol / mol creat	Detection (%)	GEV model parameters			Quantiles (p') delimiting 95% population				Normal Conc. Mmol / mol creat [39]	Origin / Disease [39]	Pathogenic Conc. mmol / mol creat	F_{global} (from model)
							From model		From data					
#	Name			*Location*	*Scale*	*Shape*	*Q2.5*	*Q97.5*	*Q2.5*	*Q97.5*				
1	1-Methyladenosine		0	-	-	-	-	-	-	-	0	Other IEMs	n. a.	-
2	1-Methylguanidine	98	93.3	119.5338	15.5537	-0.1618	-	162	-	160	0	Bacterial contam. [1]	-	-
3	1-Methylhistidine	380	0	-	-	-	-	-	-	-	0	Propionic acidemia	> 40	-
												Maple Sirup Urine disease	> 31	
4	1-Methylhydantoin	49	82.7	69.5583	17.2163	0.0558	-	136	-	138	0	Bacterial contam. [1]	-	-
5	1-Methylnicotinamide	46	63.9	56.5189	10.1029	0.3155	-	113	-	100	0.26 - 0.3	Bacterial contam. [1]	> 2.3	-
6	1,3-Dimethyluric acid	13	37	16.212	3.1027	0.275	-	28	-	28	0	Bacterial contam. [1]	-	-
7	2-Furoylglycine	39	0	-	-	-	-	-	-	-	0	Nutrition mother [2]	-	-
8	2-Hydroxy-4-methylvaleric acid	37	0	-	-	-	-	-	-	-	0	Maple Sirup Urine disease	> 50	-
9	2-Hydroxyisovaleric acid	4	2.6	3.9923	0.1973	0.6245	-	4	-	2	0 - 4	Maple Sirup Urine disease	850 - 3600	8.46e-08
10	2-Hydroxyphenylacetic acid	10	0	-	-	-	-	-	-	-	0	Phenylketonuria	50 - 2000	-
11	2-Ketobutiric acid	54	0	-	-	-	-	-	-	-	0	Methionine malabsortion	50 - 200	-
12	2-Methylsuccinic acid	34	2.0	-	-	-	-	-	-	-	0	Ethylmalonic encephalopathy	100 - 300	-
												Isovaleric acidemia	60 - 600	
												Short Chain Acyl-CoA dehydrogenase deficiency	20 - 60	
												Glutaric aciduria type II	> 60	
												Other IEMs	n. a.	

13	2-Oxoglutaric acid	160	10.9	185.1894	27.9418	0.5398	-	232	-	240	160 - 590	3-Methyl-crotonyl-glycinuria	600 - 2000	1.61e-03
14	2-Oxoisocaproic acid	5	37.2	6.7935	1.7548	0.0052	-	11	-	12	0-2	Maple Sirup Urine disease	20 - 4400	2.29e-04
15	2-Oxoisovaleric acid	4	1.5	-	-	-	-	-	-	-	0	Maple Sirup Urine disease	300 - 800	-
												Other IEMs	n. a.	
16	3-Aminoisobutyric acid	300	13.6	334.4619	36.8205	0.4643	-	420	-	445	300 - 390	Methylmalonic aciduria	400 - 1200	3.19e-02
17	3-Hydroxy-3-methylglutaric acid	110	4.2	110.8602	2.9915	0.5407	-	110	-	110	0 - 110	3-Hydroxy-3-Methylglutaryl-CoA-lyase deficiency	200 - 11000	1.74e-04
18	3-Hydroxybutyric acid	97	7.7	122.6865	24.6908	0.1571	-	145	-	150	0.5 - 9.8	β-Ketothiolase deficiency	50 - 200	>7.14e-02
												Fructose-1,6-biphosphatase deficiency	4 - 48	>7.14e-02
												Glutaric aciduria type I	200 - 800	5.38e-03
												Isovaleric acidemia	200 - 1000	5.38e-03
												Ketosis	100 - 5000	6.66e-02
												Propionic acidemia	100 - 5000	6.66e-02
												Long Chain 3-HydroxyacylCoA dehydrogenase deficiency	100 - 5000	6.66e-02
												Maple Sirup Urine disease	200 - 1000	5.38e-03
												Pyruvate Carboxylase deficiency	200 - 600	5.38e-03
19	3-Hydroxyglutaric acid	41	0.4	-	-	-	-	-	-	-	0	Glutaric aciduria type I	60 - 3000	-
												Glutaric aciduria type I (low excretor)	10 - 100	
												Glutaric aciduria type I (non-excretor)	10 - 100	

												Glutaric aciduria type II	0 - 200	
												Other IEMs	n. a.	
20	3-Hydroxyisovaleric acid	18	0.4	-	-	-	-	-	-	-	0	β-Ketothiolase deficiency	100 - 1000	-
												3-Methyl-crotonyl-glycinuria	1700-59000	
												Glutaric aciduria type II late onset	100 - 500	
												Isovaleric acidemia	100 - 2000	
												3-Hydroxy-3-Methylglutaryl-CoA-lyase deficiency	100 - 4000	
												3-Methyl-glutaconic aciduria	50-2800	
												Biotinidase deficiency	50 - 5000	
21	3-Hydroxypropionic acid	93	0.4	-	-	-	-	-	-	-	0	β-Ketothiolase deficiency	20 - 2000	-
												Lactic acidemia	20 - 1000	
												Biotinidase deficiency	20 - 500	
												Propionic acidemia	20 - 2000	
												Other IEMs	n. a.	
22	3-Hydroxyvaleric acid	6	19.7	8.9736	2.1144	0.0509	-	14	-	14	6 - 8	MMA Cbl A deficiency	2 - 1200	2.07e-01
												Propionic acidemia	4 - 1200	2.07e-01
23	3-Methyl-2-oxovaleric acid	19	0	-	-	-	-	-	-	-	0	Maple Sirup Urine disease	500 - 2500	-
												Asphyxia	10 - 50	
24	3-Methylcrotonglycine	60	0	-	-	-	-	-	-	-	0	β-Ketothiolase deficiency	11 - 500	-
												3-Hydroxy-3-Methylglutaryl-CoA-lyase deficiency	2 - 450	

												3-Methyl-crotonyl-glycinuria	10 - 1000	
												Biotinidase deficiency	5 - 50	
25	3-Methylglutaconic acid	17	0.9	-	-	-	-	-	-	-	0	3-Hydroxy-3-Methylglutaryl-CoA-lyase deficiency	60 - 10000	-
												3-Methyl-glutaconic aciduria	30 - 300	
												β-Ketothiolase deficiency	11 - 500	
26	3-Phenyllactic	85	0	-	-	-	-	-	-	-	0	Phenylketonuria	200 - 1000	-
27	4-Aminobutyric acid	17	13.1	19.2461	3.0042	0.5023	-	26	-	27	17 - 20	GABA transaminase deficiency	40 - 100	>1.24e-01
28	4-Aminohippuric acid	1700	0	-	-	-	-	-	-	-	0	Other IEMs	n. a.	-
29	4-Ethylphenol	8	0.2	-	-	-	-	-	-	-	0	Nutrition mother [1]	-	-
30	4-Hydroxyhippuric acid	980	0	-	-	-	-	-	-	-	0	Chemical contamination (parabens) [1]	-	-
31	4-Hydroxyphenylacetic acid	36	7.9	43.8161	10.2794	0.8775	-	58	-	64	36 - 69	Tyrosinemia type I	140 - 500	5.83e-03
												Tyrosinemia type II	50 - 200	3.51e-02
												Tyrosinemia type III	20 - 200	>7.62e-02
												Transient newborn tyrosinemia	50 - 200	3.51e-02
32	4-Hydroxyphenyllactic acid	470	0.2	-	-	-	-	-	-	-	0	Phenylketonuria	50 - 1000	-
												Tyrosinemia type I	100 - 5000	
												Tyrosinemia type III	20 - 2300	
33	4-Hydroxyphenylpyruvic acid	45	0.2	-	-	-	-	-	-	-	0	Tyrosinemia type I	140 - 2000	-
												Tyrosinemia type II	50 - 100	
												Tyrosinemia type III	20 - 2300	
												Transient newborn tyrosinemia	50 - 100	

34	4-Pyridoxic acid	6	68.3	6.4308	0.7417	0.6655	-	15	-	11	0	Bacterial contam. [1]	-	-
35	5-Aminolevulinic acid	3	3.8	4.1747	1.5088	0.5449	-	4	-	4	0.3	Tyrosinemia type I	20 - 200	1.49e-05
36	5-aminopentanoic acid	170	0	-	-	-	-	-	-	-	0	Bacterial contami. [1,3]	-	-
37	Acetic acid	10	99.6	40.5941	28.6368	0.4854	12	332	13	240	2.5 - 106	Phenylketonuria	> 300	
												Other IEMs	n. a.	-
38	Acetoacetic acid	5	99.3	11.5112	4.5998	0.01	5	29	5	28	5 - 28	3-Hydroxy-3-Methylglutaryl-CoA-lyase deficiency	> 28	-
												β-Ketothiolase deficiency	50 - 1000	-
												Fructose-1,6-biphosphatase deficiency	100 - 5000	-
												Ketosis	50 - 2000	0
												Propionic acidemia	50 - 5000	0
39	Acetoine	9	0	-	-	-	-	-	-	-	0	Chemical & bacterial contaminant [4]	-	-
40	Acetone	14	52.1	18.1403	4.6949	0.5187	-	51	-	41	14 - 110	Pyruvate carboxylase deficiency	30 - 100	5.65e-03
												Propionic acidemia	30 - 100	5.65e-03
												Fructose-1,6-biphosphatase deficiency	30 - 100	5.65e-03
41	Adenine	13	17.7	13.8101	1.0446	0.259	-	16	-	16	13 - 20	Adenine phosphoribosyltransferase deficiency	10 - 100	9.44e-07
42	Adenosine	950		-	-	-	-	-	-	-	0	Other IEMs	n. a.	-
43	Alanine	14	98.0	48.5749	15.4701	-0.0845	21	97	23	98	27 - 350	Hartnup disease	400 - 1400	0
												Pyruvate carboxylase deficiency	700 - 1400	0
												Ethylmalonic encephalopathy	70 - 1400	0

												Lactic acidemia	-	-
44	Allantoin	19	56.2	22.416	4.0689	0.6276	-	60	-	51	0	Bacterial contam. [5,6]	n. a.	n. a.
45	Allopurinol	18	30.2	18.7797	1.1549	0.5858	-	25	-	24	18 - 23	Cosmetic and probiotic contamination [7]	30 - 100	>3.02e-01
												Drug (active principle)	30 - 300	>3.02e-01
46	Arginine	0-4800	0	-	-	-	-	-	-	-	0	Cystinuria	> 1000	-
47	Argininosuccinic acid	99	0	-	-	-	-	-	-	-	0	Argininosuccinic aciduria	1 - 4000	-
												Citrullinemia	0.1 - 200	
48	Benzoic acid	12	3.2	-	-	-	-	-	-	-	0	Chemical & Bacterial contam. [8]	-	-
49	Betaine	44	77	255.2187	102.61	-0.134	-	536	-	556	44 - 1200	Homocistinuria	1200 - 5000	
												Dimethylglycine dehydrogenase deficiency	1200 - 5000	
												Drug (active principle)	1200 - 5000	-
50	Butyric acid	36	25.6	29.5057	3.1989	0.0697	-	37	-	37	0	Bacterial contam. [9,10]	-	-
51	Caffeine	8	8.3	8.8787	1.1222	0.3899	-	10	-	10	0	Nutrition mother [11]	-	-
52	Choline	94	0	-	-	-	-	-	-	-	0	Nutrition mother [1]	-	-
53	Citraconic acid	86	0	-	-	-	-	-	-	-	0	Nutrition mother [1,12]	-	-
54	Citric acid	46	60.8	85.1302	42.3419	0.4832	-	398	-	300	46 - 1400	3-Methylg-lutaconic aciduria	500 - 25000	1.58e-02
55	Citrulline	780	0	-	-	-	-	-	-	-	0	Citrullinemia	781 - 5000	-
												GABA transaminase deficiency	781 - 2000	
												Other IEMs	n. a.	
56	Creatine (CRE)	50	73.7	270.1886	191.6025	0.0375	-	962	-	901	1 - 1.8	Creatine transport deficiency	CRE/CRN > 2	-
57	Creatinine (CRN)	-	100	6.2413	3.9766	-0.136	1	18	2	17	1	Creatine transport deficiency	CRE/CRN > 2	-
58	Cystine	75	6.6	86.5746	9.7811	0.4668	-	94	-	95	0 - 75	Cystinuria	700 - 10000	4.01e-05

59	Cytosine	9	0	-	-	-	-	-	-	-	1.1 - 10.7	Other IEMs	n. a.	-
60	D-Galactonic acid	130	0.2	-	-	-	-	-	-	-	0	Galactosemia	> 5	-
												Other IEMs	n. a.	
61	D-Galactose	85	34.7	154.2749	67.3716	0.3394	-	434	-	420	85 - 140	Citrullinemia	600 - 12000	3.47e-01
												Galactosemia type III	150 - 800	>3.47e-01
												Galactosemia type II	400 - 1200	3.09e-02
												Galactosemia type I	600 - 12000	1.06e-02
												Fanconi-Bickel syndrome	> 85	-
62	D-Gluconic acid	190	0	-	-	-	-	-	-	-	0	Bacterial contam. [13,14]	-	-
63	D-Glucose	76	92.6	164.8134	56.4634	0.1581	-	439	-	412	76 - 800	Glucose-galactose malabsorption	800 - 16000	3.47e-01
												Fanconi-Bickel syndrome	800 - 16000	>3.47e-01
64	D-Lactose	96	68.3	199.3838	92.6355	0.1854	-	619	-	591	96 - 850	Lactose intolerance	100 - 2000	3.09e-02
65	D-Mandelic acid	4	0.4	-	-	-	-	-	-	-	0 - 4	Phenylketonuria	8 - 135	-
66	D-Mannitol	430	1.5	-	-	-	-	-	-	-	0	Drug (excipient) [15]	10 - 100	-
67	D-Mannose	20	45.7	28.6174	9.0794	0.3718	-	73	-	66	0	Bacterial contam. [1]	-	-
68	D-Panthenol	19	0.9	-	-	-	-	-	-	-	0	Drug (excipient) [16]	10 - 100	-
69	D-Xylose	840	0	-	-	-	-	-	-	-	0	Other IEMs	n. a.	-
70	Dihydrothymine	430	2.4	-	-	-	-	-	-	-	0	Dihydropyriminidase deficiency	800 - 4000	-
												Other IEMs	n. a.	
71	Dihydrouracil	130	0	-	-	-	-	-	-	-	0	Dihydropyriminidase deficiency	> 0	-
72	Dimethylamine	8	100	91.8363	22.2719	-0.2457	58	146	59	140	20 - 60	Chemical contam. & Nutrition mother [17,18]	-	-
73	DL-Alloisoleucine	47	0	-	-	-	-	-	-	-	0	Bacterial contam. [1]	-	-
74	DL-Kyneurin	790	0	-	-	-	-	-	-	-	0	Kynureninase deficiency	10- 100	-
75	DL-Tyrosine	43	0	-	-	-	-	-	-	-	0	Methylmalonic aciduria	150 - 15500	-

												Tyrosinemia type I	> 150	
												Hartnup disease	50 - 500	
76	E-Glutaconic acid	130	0	-	-	-	-	-	-	-	0	Glutaric aciduria type I	2 - 360	-
												Glutaric aciduria type I (low excretor)	2- 360	
												Glutaric aciduria type I (non-excretor)	2- 360	
												Glutaric aciduria type II	2 - 360	
77	Ethanol	840	0	-	-	-	-	-	-	-	0	Nutrition mother [19]	-	-
78	Ethylmalonic acid	20	27.3	21.5563	1.984	0.4114	-	29	-	29	20 - 40	Ethylmalonic encephalopathy	50 - 500	2.34e-03
												Short Chain Acyl-CoA dehydrogenase deficiency	180 - 1150	4.92e-05
												Glutaric aciduria type II	50 - 1200	2.34e-03
												Glutaric aciduria type II (late onset)	50 - 1200	2.34e-03
79	Formic acid	92	32.8	121.4924	31.9467	0.6439	-	313	-	290	20 - 200	Chemical contam. [20,21]	-	-
80	Fumaric acid	2	92.8	5.845	2.9225	0.1208	-	19	-	18	2 - 40	2-Ketoadipic acidemia	100 - 1000	1.82e-06
												Fumaric aciduria	3000 - 4000	0
												Ethylmalonic encephalopathy	> 100	
81	Galactitol	300	0	-	-	-	-	-	-	-	0	Galactosemia	30 - 90000	-
												Fanconi-Bickel syndrome	5 - 500	
82	Glutamic acid	330	0.4	-	-	-	-	-	-	-	0	Dicarboxylic aminoaciduria	50 - 1400	-
												Other IEMs	n. a.	
83	Glutamine	830	0	-	-	-	-	-	-	-	0	Hartnup disease	300 - 3000	-
												Other IEMs	n. a.	

84	Glutaric acid	67	0	-	-	-	-	-	-	-	0	3-Hydroxy-3-Methylglutaryl-CoA-lyase deficiency	2 - 100	-
												Glutaric acidemia type I	500 - 12000	
												Glutaric acidemia type I (low excretor)	1-200	
												Glutaric acidemia type II	500 - 12000	
												Glutaric acidemia type II (late onset)	500 - 12000	
												Glutaric acidemia type III	100 - 500	
85	Glycerol	790	0	-	-	-	-	-	-	-	0	Fructose intolerance	220 - 2000	-
86	Glycine	30	98.7	262.4517	130.1257	-0.0027	70	737	78	749	87 - 1900	Drug (active principle & excipient)	1000 - 3000	5.21e-01
												Isovaleric acidemia	500 - 5000	3.26e-03
												Propionic acidemia	2000 - 2500	3.26e-03
												Transient immaturity of transporters	2000 - 6000	3.26e-06
												Ornithine transcarbamilase deficiency	2000 - 5000	7.91e-01
												Arginosuccinic aciduria	2000 - 5000	1.23e-03
												MMA Cbl A deficiency	2000 - 5000	3.26e-03
87	Glycolic acid	190	30.9	220.487	30.864	0.2947	-	333	-	329	190 - 480	Hyperoxaluria type II	> 500	>3.09e-01
88	Guanidinoacetic acid	93	27.7	106.2827	12.6332	0.1353	-	141	-	140	93 - 190	Argininemia	> 200	-
												Guanidinoacetate Methyltransferase deficiency	> 200	-
89	Hippuric acid	49	5.3	53.1678	3.3695	0.2838	-	55	-	54	49 - 150	Argininemia	200 - 5000	-
												Argininosuccinic aciduria	1000 - 15000	1.01e-08
												Citrullinemia	200 - 50000	5.01e-02
												Transient infantile liver failure	1000 - 15000	1.01e-08

												MMA Cbl A deficiency	1000 - 15000	1.01e-08
												Ornithine transcarbamilase deficiency	300 - 10000	>5.28e-02
												Propionic acidemia	1000 - 15000	1.01e-08
												Drug (excipient) [22]	500 - 50000	1.35e-07
90	Imidazole	38	0	-	-	-	-	-	-	-	9	Chemical & bacterial contam. [1]	-	-
91	Inosine	17	0.4	-	-	-	-	-	-	-	0	Methylmalonic aciduria	150 - 15500	-
												Other IEMs	n. a.	
92	Isobutyrylglycine	29	0	-	-	-	-	-	-	-	0	Glutaric aciduria type II	5 - 200	-
												Other IEMs	n. a.	
93	Isopropanol	9	4.5	13.5872	4.7241	0.6861	-	15	-	15	0 - 9	Chemical contam. [23]	-	>4.53e-02
94	L-Ascorbic acid	270	0	-	-	-	-	-	-	0	0	Drug (active principle & excipient) [24]	-	-
95	L-Citramalic acid	560	0	-	-	-	-	-	-	0	0 - 100	n. d.	-	-
96	L-Fucose	300	20.5	366.7539	74.63	0.3979	-	601	-	605	0	Bacterial contam. [1]	-	-
97	L-Homocystine	170	0	-	-	-	-	-	-	0	0 - 1	Homocystinuria	12 - 250	-
98	L-Isoleucine	47	0	-	-	-	-	-	-	0	0	Hartnup disease	50 - 500	-
												Maple Sirup Urine disease	50 - 500	
99	L-Pyroglutamic acid	5	23.2	40.3368	13.3198	-0.1977	-	64	-	64	5 - 44	Drug (active principle & excipient) [25,26]	50 - >500	2.03e-01
												MMA Cbl A deficiency	100 - 1000	4.03e-06
												Ornithine transcarbamilase deficiency	50 - 1000	8.54e-02
												Propionic acidemia	50 - 100	2.03e-01
												Transient immaturity of transporters	50 - 500	8.54e-02
100	L-Threonic acid	180	0.4	-	-	-	-	-	-	-	0 - 100	Bacterial contam. [1,27]	100 - 500	-

101	L-Tryptophan	49	0	-	-	-	-	-	-	-	0	Hartnup disease	80 - 240	-
												Other IEMs	n. a.	
102	Lactic acid	45	90	81.522	32.3222	0.3617	-	317	-	250	45 - 410	3-Methyl-glutaconic aciduria type IV	500 - 1000	2.90e-02
												Biotinidase deficiency	500 - 75000	2.90e-02
												Pyruvate decarboxylase deficiency	500 - 3000	2.90e-02
												Lactic acidemia	500 - 3000	2.90e-02
												Ethylmalonic encephalopathy	400 - 3000	
												Asphyxia	400 - 1000	4.03e-02
												Bacterial contam. [28]	100 - 7500	4.03e-01
103	Leucine	13	64.7	16.3284	2.6238	-0.0847	-	24	-	23	13 - 40	Maple Sirup Urine disease	80 - 240	0
												Hartnup disease	50 - 200	0
104	Maleic acid	7	22.3	8.7323	2.2071	0.5818	-	17	-	18	2 - 40	Bacterial contam. [1]	-	-
105	Malic acid	81	4.2	167.1373	40.5147	-0.1954	-	164	-	160	81 - 250	2-Ketoadipic acidemia	500 - 1500	-
106	Methanol	91	11.6	108.0949	15.5829	0.3429	-	131	-	130		Bacterial contam. [29]	-	-
107	Methionine	13	0.2	-	-	-	-	-	-	-	0	Homocystinuria	25 - 200	-
												Other IEMs	n. a.	
108	Methylmalonic acid	10	12.7	11.6077	1.9505	0.3023	-	15	-	15	10 - 20	Isovaleric aciduria	50 - 500	2.00e-04
												MMA Cbl A deficiency	150 - 15500	4.13e-06
109	Myo-Inositol	2500	0.7	-	-	-	-	-	-	-		Transketolase deficiency	-	-
110	N-Acetylaspartic acid	48	1.8	-	-	-	-	-	-	-	0	Canavan disease	100 - 10000	-
111	N-Acetylglutamate	42	0	-	-	-	-	-	-	-	0	Aminoacylase I deficiency	50 - 100	-
112	N-Acetylphenylalanine	87	0	-	-	-	-	-	-	-	0	Phenylketonuria	100 - 500	-
113	N-Acetyltyrosine	1100	0	-	-	-	-	-	-	-	0	Tyrosinemia type I	> 200	-
												Tyrosinemia type II	> 200	

114	N-Isovaleroylglycine	2	43.1	2.8116	0.8636	-0.1523	-	5	-	5	2 - 5	Ethylmalonic encephalopathy	5 - 150	1.61e-02
												Glutaric aciduria type II	5 - 1000	1.61e-02
												Isovaleric aciduria	10 - 9000	0
115	N,N-Dimethylglycine	15	97.5	40.3539	15.4228	-0.0359	-	93	15	94	15 - 220	Dimethylglycine dehydrogenase deficiency	300 - 600	5.91e-12
116	Neopterin	17	0	-	-	-	-	-	-	-	0 - 2	Other IEMs	> 20	-
117	Orotic acid	5	0.4	-	-	-	-	-	-	-	0	Argininemia	10 - 1000	-
												Argininosuccinic aciduria	2 - 500	
												Citrullinemia	5 - 600	
												Ornithine transcarbamilase deficiency	10 - 1300	
												Orotic aciduria	1400 - 5600	
												Other IEMs	n. a.	
118	Oxaloacetic acid	44	53.6	69.8169	23.9507	0.3212	-	190	-	180	0 - 5	Other IEMs	n. a.	-
119	Oxypurinol	19	40.2	20.8088	1.9907	0.3055	-	29	-	29	19 - 37	Drug (excipient) [31]	10 - 300	>4.02e-01
120	Pantothenic acid	20	71.3	21.9973	2.1004	0.2011	-	32	-	30	20 - 50	Drug (excipient) [1]	50 - 1000	>6.89e-01
121	Paracetamol	50	0	-	-	-	-	-	-	-	0	Drug (active princ.) [32]	-	-
122	Paracetamol-glucuronide	1500	0	-	-	-	-	-	-	-	0	Drug (active princ.) [33]	-	-
123	Phenylacetic acid	470	0	-	-	-	-	-	-	-	0	Phenylketonuria	500 - 5000	-
124	Phenylalanine	120	0	-	-	-	-	-	-	-	0	Phenylketonuria	50 - 1000	-
												Other IEMs	n. a.	
125	Phenylpyruvic acid	85	0	-	-	-	-	-	-	-	0	Phenylketonuria	300 - 1000	-
126	Pimelic acid	74	0	-	-	-	-	-	-	-	0	Other IEMs	n. a.	-
127	Proline betaine	24	47.3	34.9607	9.5305	0.0797	-	66	-	64	0	Nutrition mother [34]	-	-
128	Propionic acid	76	7.9	85.687	12.5668	0.8354	-	97	-	96	0 - 76	Propionic acidemia	n. a.	n.a.
129	Propionylglycine	2	0.2	-	-	-	-	-	-	-	0	Medium Chain Acyl CoA deficiency	1 - 90	-

												Propionic acidemia	4 - 450	
												Methylmalonic aciduria	5 - 450	
130	Propylene glycol	45	51.0	77.7727	32.4224	0.3924	-	256	-	219	45 - 1200	Chemical contam. [1]	-	-
131	Pyrocatechol	170	0	-	-	-	-	-	-	-	0 - 15	Other IEMs	-	-
132	Pyruvic acid	13	77.1	16.5817	3.3106	0.145	-	31	-	30	13 - 41	3-Methyl-glutaconic aciduria	50 - 200	1.54e-03
133	Quinolinic acid	38	0	-	-	-	-	-	-	-	0	Bacterial contam. [35]	-	-
134	Sarcosine	5	58.0	5.2745	0.5865	0.8539	-	14	-	9	5 - 25	Glutaric aciduria type II	25 - 100	1.54e-03
												Sarcosinemia	100 - 5000	4.56e-01
												Nutrition of the mother	30 - 300	4.56e-01
135	Succinic acid	8	88.4	17.8749	8.5641	0.4197	-	87	-	67	9 - 360	2-Ketoadipic acidemia	400 - 1200	7.14e-04
												3-Methyl-glutaconic aciduria type II	400 - 1200	7.14e-04
												Ethylmalonic encephalopathy	400 - 1200	7.14e-04
												Canavan disease	n. a.	n. a.
												Pyruvate carboxylase deficiency	400 - 1200	7.14e-04
												Fumaric aciduria	400 - 1200	7.14e-04
136	Succinylacetone	410	0	-	-	-	-	-	-	-	0 - 10	Tyrosinemia type I	> 10	-
137	Syringic acid	10	10.5	10.9365	1.2741	0.4834	-	13	-	13		Bacterial contam & Nutrition mother [36,37]	-	-
138	Tartaric acid	45	2.2	-	-	-	-	-	-	-	0 - 60	Other IEMs	-	-
139	Taurine	250	29.3	313.7225	72.0296	0.609	-	689	-	679	250 - 910	Other IEMs	900 - 2000	1.42e-02
140	Theobromine	81	1.1	-	-	-	-	-	-	-		Nutrition mother [38]	-	-
141	Thymine	4	1.5	-	-	-	-	-	-	-	0	Dihydropiriminidase deficiency	2 - 40	-
												Dihydropyrimidine Dehydrogenase deficiency	20 - 80	

142	Thymol	170	0	-	-	-	-	-	-	-	0	Chemical contamination & drug (excipient)	-	-
143	Tiglylglycine	74	0	-	-	-	-	-	-	-	0	β-Ketothiolase deficiency	20 - 1500	-
												Propionic acidemia	20 - 200	
												Other IEMs	n. a.	
144	Trigonelline	33	0	-	-	-	-	-	-	-	0	Nutrition mother [1]	-	-
145	Trimethylamine	2	13.6	2.1468	0.4187	0.8613	-	3	-	4	2 - 6	Trimethylaminuria	10 - 300	4.25e-03
146	Tyramine	860	0	-	-	-	-	-	-	-	0	Dihydropyriminidase deficiency	> 400	-
147	Uracil	8	26.3	11.4359	3.1893	0.1877	-	20	-	22	8 - 110	Argininemia	> 100	4.67e-03
												Argininosuccinic aciduria	100 - 500	4.37e-03
												Dihydropyriminidase deficiency	100 - 500	4.37e-03
												Dihydropyrimidine Dehydrogenase deficiency	100 - 150	4.37e-03
												Citrullinemia	100 - 700	
												Ornithine transcarbamilase deficiency	100 - 500	4.67e-03
												Tyrosinemia type I	100 - 200	4.37e-03
148	Uridine	15	0	-	-	-	-	-	-	-	0	Ornithine transcarbamilase deficiency	5 - 50	-
												Citrullinemia	5 - 50	-
149	Valine	5	66.5	8.0124	2.7203	0.1213	-	19	-	18	5 - 24	Maple Syrup Urine disease	20 - 50	6.30e-01
												Hartnup disease	40 - 500	4.39e-04
150	Xanthurenic acid	18	0	-	-	-	-	-	-	-	0	Kynureninase deficiency	50 - 200	-

References to Table S2

[1] Bouatra S, Aziat F, Mandal R, Guo AC, Wilson MR, Knox C, et al. The human urine metabolome. PloS one. 2013;8(9):e73076. Epub 2013/09/12. doi: 10.1371/journal.pone.0073076. PubMed PMID: 24023812; PubMed Central PMCID: PMCPMC3762851.

[2] Pettersen JE, Jellum E. The identification and metabolic origin of 2-furoylglycine and 2,5-furandicarboxylic acid in human urine. Clinica chimica acta; international journal of clinical chemistry. 1972;41:199-207. Epub 1972/10/01. doi: 10.1016/0009-8981(72)90512-8. PubMed PMID: 4630229.

[3] Fothergill JC, Guest JR. Catabolism of L-lysine by Pseudomonas aeruginosa. Journal of general microbiology. 1977;99(1):139-55. Epub 1977/03/01. doi: 10.1099/00221287-99-1-139. PubMed PMID: 405455.

[4] Curwin BD, Deddens JA, McKernan LT. Flavoring exposure in food manufacturing. J Expo Sci Environ Epidemiol. 2015;25(3):324-33. Epub 07/23. doi: 10.1038/jes.2014.52. PubMed PMID: 25052692.

[5] Goelzer A, Bekkal Brikci F, Martin-Verstraete I, Noirot P, Bessieres P, Aymerich S, et al. Reconstruction and analysis of the genetic and metabolic regulatory networks of the central metabolism of Bacillus subtilis. BMC systems biology. 2008;2:20. Epub 2008/02/28. doi: 10.1186/1752-0509-2-20. PubMed PMID: 18302748; PubMed Central PMCID: PMCPMC2311275.

[6] Navone L, Casati P, Licona-Cassani C, Marcellin E, Nielsen LK, Rodriguez E, et al. Allantoin catabolism influences the production of antibiotics in Streptomyces coelicolor. Applied microbiology and biotechnology. 2014;98(1):351-60. Epub 2013/12/03. doi: 10.1007/s00253-013-5372-1. PubMed PMID: 24292080.

[7] Knox C, Law V, Jewison T, Liu P, Ly S, Frolkis A, et al. DrugBank 3.0: a comprehensive resource for 'omics' research on drugs. Nucleic acids research. 2011;39(Database issue):D1035-41. Epub 2010/11/10. doi: 10.1093/nar/gkq1126. PubMed PMID: 21059682; PubMed Central PMCID: PMCPMC3013709.

[8] Beloborodova N, Bairamov I, Olenin A, Shubina V, Teplova V, Fedotcheva N. Effect of phenolic acids of microbial origin on production of reactive oxygen species in mitochondria and neutrophils. Journal of biomedical science. 2012;19:89. Epub 2012/10/16. doi: 10.1186/1423-0127-19-89. PubMed PMID: 23061754; PubMed Central PMCID: PMCPMC3503878.

[9] Duncan SH, Barcenilla A, Stewart CS, Pryde SE, Flint HJ. Acetate utilization and butyryl coenzyme A (CoA):acetate-CoA transferase in butyrate-producing bacteria from the human large intestine. Applied and environmental microbiology. 2002;68(10):5186-90. Epub 2002/09/27. doi: 10.1128/aem.68.10.5186-5190.2002. PubMed PMID: 12324374; PubMed Central PMCID: PMCPMC126392.

[10] Riviere A, Selak M, Lantin D, Leroy F, De Vuyst L. Bifidobacteria and Butyrate-Producing Colon Bacteria: Importance and Strategies for Their Stimulation in the Human Gut. Frontiers in Microbiology. 2016;7:979. Epub 2016/07/23. doi: 10.3389/fmicb.2016.00979. PubMed PMID: 27446020; PubMed Central PMCID: PMCPMC4923077.

[11] Lamarine RJ. Selected health and behavioral effects related to the use of caffeine. Journal of community health. 1994;19(6):449-66. Epub 1994/12/01. PubMed PMID: 7844249.

[12] The Merck Index: An Encyclopedia of Chemicals, Drugs, and Biologicals 1996.

[13] Kim S, Chen J, Cheng T, Gindulyte A, He J, He S, et al. PubChem 2019 update: improved access to chemical data. Nucleic acids research. 2019;47(D1):D1102-D9. doi: 10.1093/nar/gky1033. PubMed PMID: 30371825.

[14] National Center for Biotechnology Information. PubChem Database https://pubchem.ncbi.nlm.nih.gov/compound/.

[15] Varzakas TL, Athanasios; Anestis, Styliano. Sweeteners: Nutritional Aspects, Applications, and Production Technology2012.

[16] Ebner F, Heller A, Rippke F, Tausch I. Topical use of dexpanthenol in skin disorders. American journal of clinical dermatology. 2002;3(6):427-33. Epub 2002/07/13. doi: 10.2165/00128071-200203060-00005. PubMed PMID: 12113650.

[17] Steinhagen WH, Swenberg JA, Barrow CS. Acute inhalation toxicity and sensory irritation of dimethylamine. American Industrial Hygiene Association journal. 1982;43(6):411-7. Epub 1982/06/01. doi: 10.1080/15298668291409956. PubMed PMID: 7113921.

[18] Neurath GB, Dunger M, Pein FG, Ambrosius D, Schreiber O. Primary and secondary amines in the human environment. Food and cosmetics toxicology. 1977;15(4):275-82. Epub 1977/08/01. PubMed PMID: 590888.

[19] Bhuvaneswar CG, Chang G, Epstein LA, Stern TA. Alcohol use during pregnancy: prevalence and impact. Prim Care Companion J Clin Psychiatry. 2007;9(6):455-60. doi: 10.4088/pcc.v09n0608. PubMed PMID: 18185825.

[20] Liesivuori J, Savolainen H. Methanol and formic acid toxicity: biochemical mechanisms. Pharmacology & toxicology. 1991;69(3):157-63. Epub 1991/09/01. PubMed PMID: 1665561.

[21] Gupta A. To err is Genetics: diagnosis and management of inborn errors of metabolism (IEM). Chapter. 2007;32:415-23.

[22] Knowles CO. 22.4.2 Benzyl Benzoate in Handbook of Pesticide Toxicology, Volume 3: Classes of Pesticides"1991.
[23] Vicas IM, Beck R. Fatal inhalational isopropyl alcohol poisoning in a neonate. Journal of toxicology Clinical toxicology. 1993;31(3):473-81. Epub 1993/01/01. PubMed PMID: 8355323.
[24] Yannai S. Dictionary of food compounds with CD-ROM: Additives, flavors, and ingredients.2004.
[25] Oberholzer VG, Wood CB, Palmer T, Harrison BM. Increased pyroglutamic acid levels in patients on artificial diets. Clinica chimica acta; international journal of clinical chemistry. 1975;62(2):299-304. Epub 1975/07/23. doi: 10.1016/0009-8981(75)90240-5. PubMed PMID: 1149292.
[26] Biff F. Palmer RJA. Metabolic Acidosis In Comprehensive Clinical Nephrology Fourth Edition ed.
[27] A.Seib M-LP. Chemistry of L-ascorbic acid related to foods. Food Chemistry. 1988;30(4):289-312.
[28] Gupta A, Dwivedi M, Mahdi AA, Khetrapal CL, Bhandari M. Broad identification of bacterial type in urinary tract infection using (1)h NMR spectroscopy. Journal of proteome research. 2012;11(3):1844-54. Epub 2012/02/02. doi: 10.1021/pr2010692. PubMed PMID: 22292465.
[29] Megarbane B, Borron SW, Baud FJ. Current recommendations for treatment of severe toxic alcohol poisonings. Intensive care medicine. 2005;31(2):189-95. Epub 2005/01/01. doi: 10.1007/s00134-004-2521-0. PubMed PMID: 15627163.
[30] Boyle L, Wamelink MMC, Salomons GS, Roos B, Pop A, Dauber A, et al. Mutations in TKT Are the Cause of a Syndrome Including Short Stature, Developmental Delay, and Congenital Heart Defects. American journal of human genetics. 2016;98(6):1235-42. doi: 10.1016/j.ajhg.2016.03.030. PubMed PMID: 27259054.
[31] Oxipurinol: alloxanthine, Oxyprim, oxypurinol. Drugs in R&D. 2004;5(3):171-5. Epub 2004/05/14. doi: 10.2165/00126839-200405030-00008. PubMed PMID: 15139781.
[32] Acetaminophen. Drugs in pregnancy and lactation: a reference guide to fetal and neonatal risk. h; :. 10th ed: Philadelphia: Wolters Kluwer Healt; 2015.
[33] Jacobs DM, Spiesser L, Garnier M, de Roo N, van Dorsten F, Hollebrands B, et al. SPE-NMR metabolite sub-profiling of urine. Analytical and bioanalytical chemistry. 2012;404(8):2349-61. Epub 2012/08/31. doi: 10.1007/s00216-012-6339-2. PubMed PMID: 22932811.
[34] Atkinson W, Downer P, Lever M, Chambers ST, George PM. Effects of orange juice and proline betaine on glycine betaine and homocysteine in healthy male subjects. European journal of nutrition. 2007;46(8):446-52. Epub 2007/12/07. doi: 10.1007/s00394-007-0684-5. PubMed PMID: 18060588.
[35] Guneral F, Bachmann C. Age-related reference values for urinary organic acids in a healthy Turkish pediatric population. Clinical Chemistry. 1994;40(6):862.
[36] Boto-Ordonez M, Urpi-Sarda M, Queipo-Ortuno MI, Tulipani S, Tinahones FJ, Andres-Lacueva C. High levels of Bifidobacteria are associated with increased levels of anthocyanin microbial metabolites: a randomized clinical trial. Food & function. 2014;5(8):1932-8. Epub 2014/06/25. doi: 10.1039/c4fo00029c. PubMed PMID: 24958563.
[37] Forester SC, Waterhouse AL. Identification of Cabernet Sauvignon anthocyanin gut microflora metabolites. Journal of agricultural and food chemistry. 2008;56(19):9299-304. Epub 2008/09/05. doi: 10.1021/jf801309n. PubMed PMID: 18767860.
[38] Craig WJ, Nguyen TT. Caffeine and Theobromine Levels in Cocoa and Carob Products. Journal of food Science. 1984;49(1):302-3.
[39] G.Frauendienst-Egge, Trefz FK. MetaGene: Metabolic & Genetic Information Center: METAGENE consortium. MetaGene: Metabolic & Genetic Information Center2017. Retrieved on 150819.

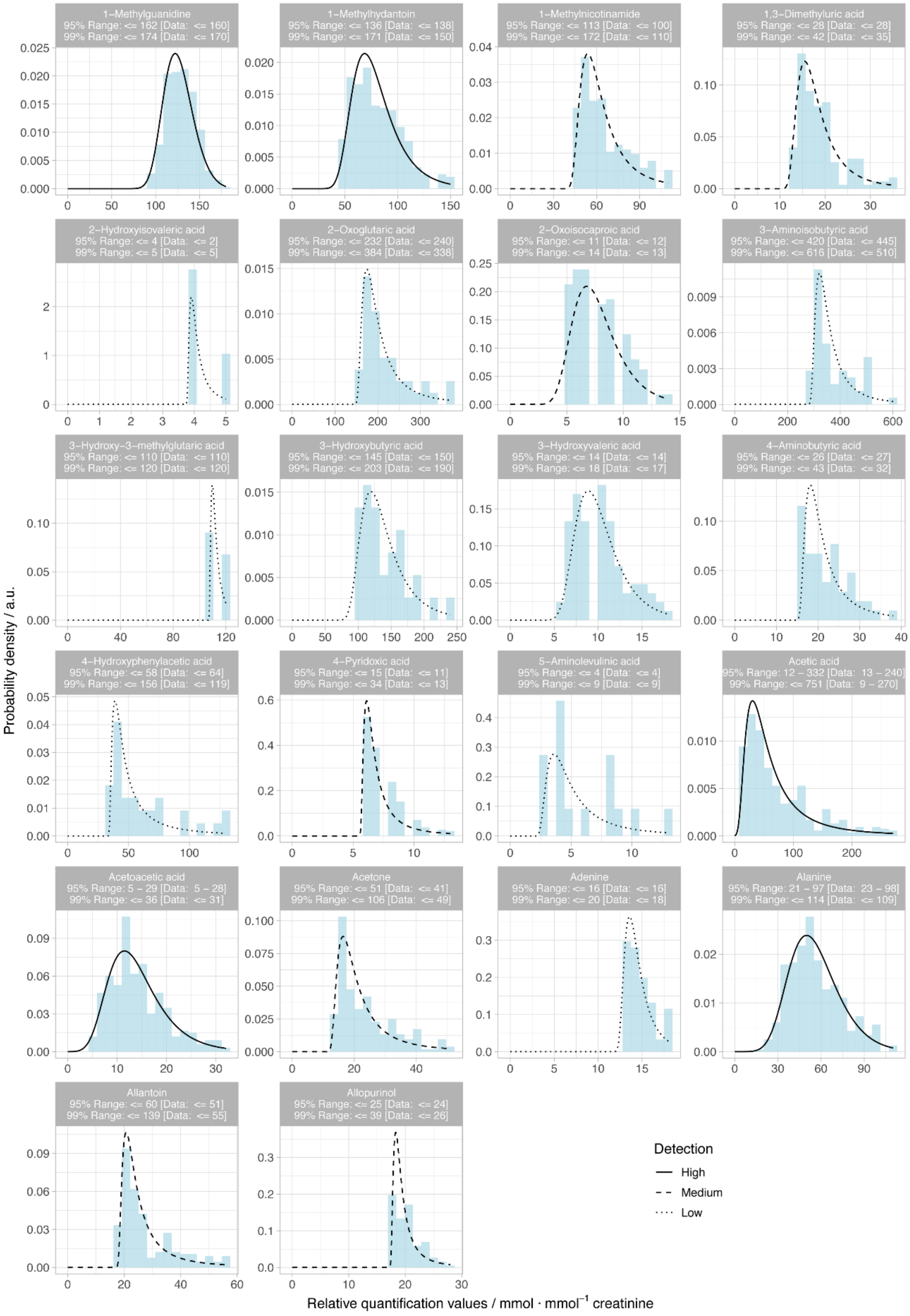

Figure S4. Distribution of probability densities for 22 out of the 66 metabolites that had a percentage of detection greater or equal 2.5%. They were used to build GEV probabilistic models. Densities calculated from models are represented as black lines with the following legend: dotted for low, dashed for intermediate and solid for high percentage of detection for the metabolite. Light blue bars plot experimental real data, where values below LOD were discarded.

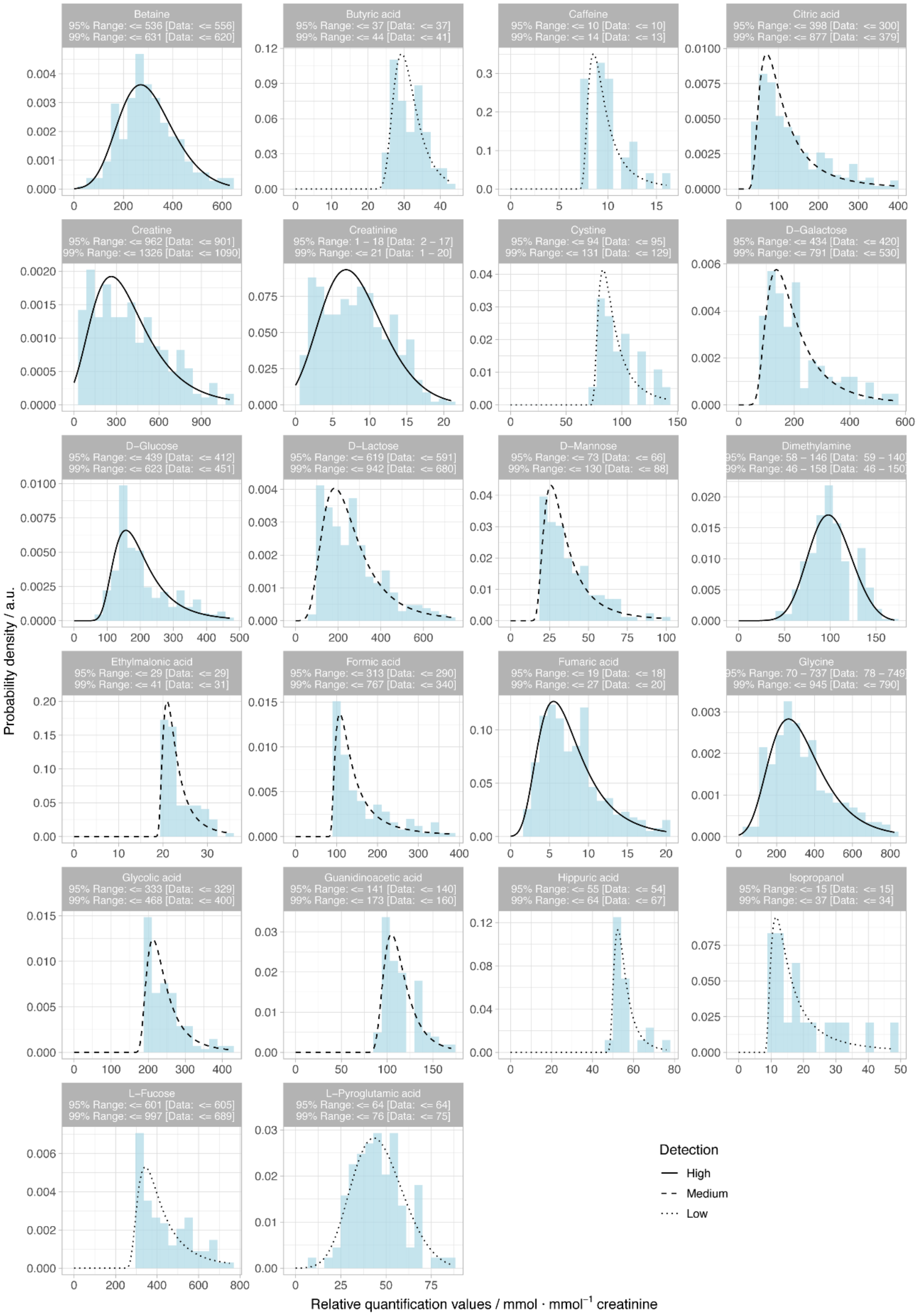

Figure S5. Distribution of probability densities for 22 out of the 66 metabolites that had a percentage of detection greater or equal 2.5%. They were used to build GEV probabilistic models. Densities calculated from models are represented as black lines with the following legend: dotted for low, dashed for intermediate and solid for high percentage of detection for the metabolite. Light blue bars plot experimental real data, where values below LOD were discarded.

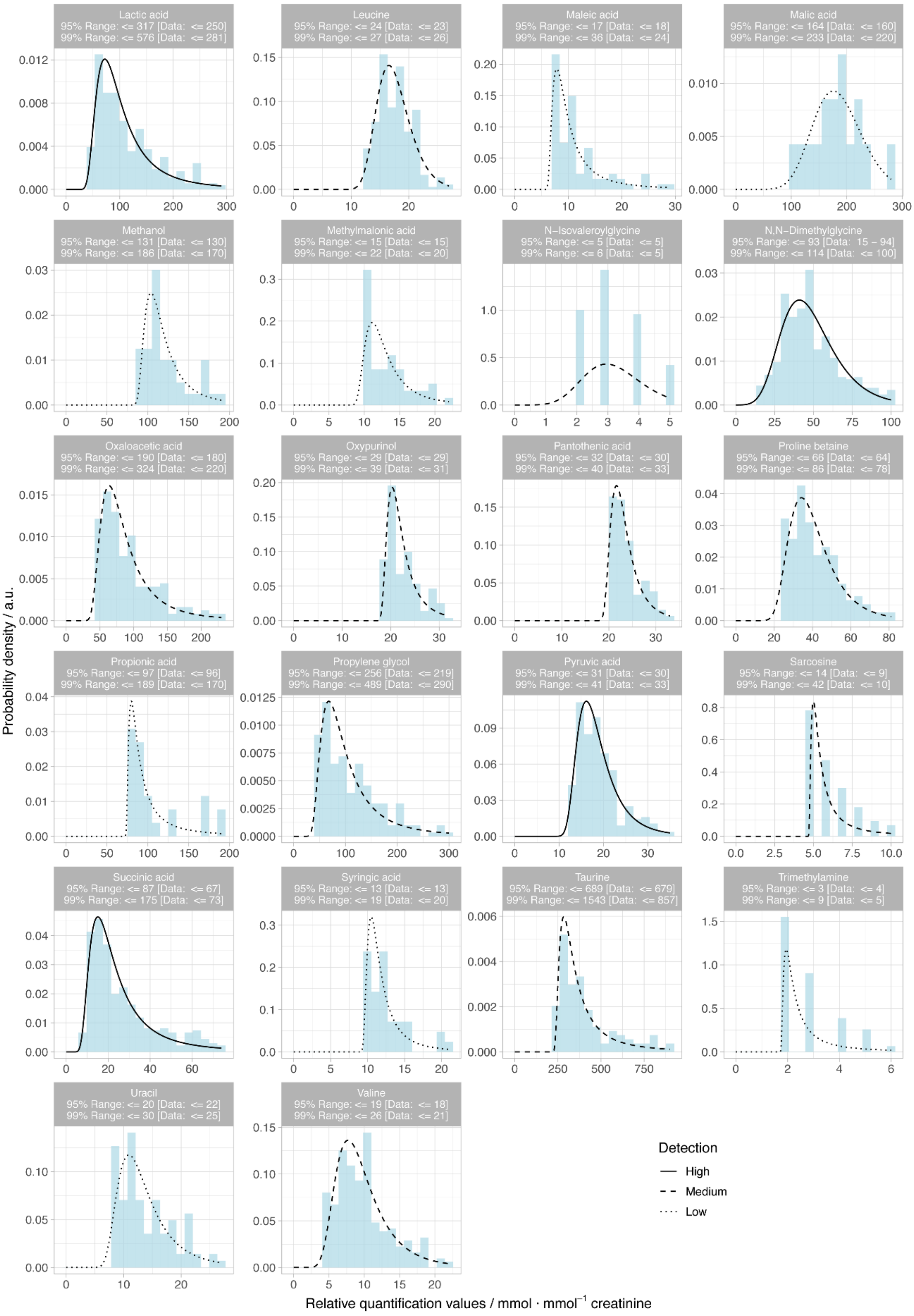

Figure S6. Distribution of probability densities for 22 out of the 66 metabolites that had a percentage of detection greater or equal 2.5%. They were used to build GEV probabilistic models. Densities calculated from models are represented as black lines with the following legend: dotted for low, dashed for intermediate and solid for high percentage of detection for the metabolite. Light blue bars plot experimental real data, where values below LOD were discarded
